# Eukaryotic translation initiation factor 4E family member nCBP facilitates the accumulation of TGB-encoding viruses by recognizing the viral coat protein in potato and tobacco

**DOI:** 10.3389/fpls.2022.946873

**Published:** 2022-08-08

**Authors:** Ruhao Chen, Manhua Yang, Zhen Tu, Fangru Xie, Jiaru Chen, Tao Luo, Xinxi Hu, Bihua Nie, Changzheng He

**Affiliations:** ^1^ERC for Germplasm Innovation and New Variety Breeding of Horticultural Crops, Key Laboratory for Vegetable Biology of Hunan Province, Hunan Agricultural University, Changsha, China; ^2^Key Laboratory of Potato Biology and Biotechnology (HZAU), Ministry of Agriculture and Rural Affairs, Key Laboratory of Horticultural Plant Biology (HZAU), Ministry of Education, Huazhong Agricultural University, Wuhan, China

**Keywords:** susceptibility genes, recessive resistance, eukaryotic translation initiation factors 4E, nCBP, TGB-encoding virus, coat protein

## Abstract

Due to their limited coding capacity, plant viruses have to depend on various host factors for successful infection of the host. Loss of function of these host factors will result in recessively inherited resistance, and therefore, these host factors are also described as susceptibility genes or recessive resistance genes. Most of the identified recessive resistance genes are members of the *eukaryotic translation initiation factors 4E* family (*eIF4E*) and its isoforms. Recently, an eIF4E-type gene, *novel cap-binding protein* (*nCBP*), was reported to be associated with the infection of several viruses encoding triple gene block proteins (TGBps) in Arabidopsis. Here, we, for the first time, report that the knockdown of *nCBP* in potato (*StnCBP*) compromises the accumulation of potato virus S (PVS) but not that of potato virus M (PVM) and potato virus X (PVX), which are three potato viruses encoding TGBps. Further assays demonstrated that StnCBP interacts with the coat proteins (CPs) of PVS and PVM but not with that of PVX, and substitution of PVS CP in the PVS infectious clone by PVM CP recovered the virus infection in *StnCBP*-silenced transgenic plants, suggesting that the recognition of PVS CP is crucial for *StnCBP*-mediated recessive resistance to PVS. Moreover, the knockdown of nCBP in *Nicotiana benthamiana* (*NbnCBP*) by virus-induced gene silencing suppressed PVX accumulation but not PVM, while NbnCBP interacted with the CPs of both PVX and PVM. Our results indicate that the *nCBP* orthologues in potato and tobacco have conserved function as in Arabidopsis in terms of recessive resistance against TGB-encoding viruses, and the interaction between nCBP and the CP of TGB-encoding virus is necessary but not sufficient to determine the function of *nCBP* as a susceptibility gene.

## Introduction

Potato (*Solanum tuberosum* L.) is currently the third most important food crop worldwide next to rice and wheat for direct human consumption and food security (Hardigan et al., 2017; van der Waals and Krüger, 2020). However, plant viruses are one of the main limiting factors leading to a loss in quality and quantity of potato (Wang et al., 2011; Rashid et al., 2021). Due to their limited coding capacity, plant viruses recruit various host factors to help their infection (Truniger and Aranda, 2009). These host factors, whose mutation confers loss of susceptibility to related viruses, are called susceptibility genes or recessive resistance genes (Truniger and Aranda, 2009; Hashimoto et al., 2016; Garcia-Ruiz, 2018; Mäkinen, 2020).

The eukaryotic translation initiation factor 4E (eIF4E) and its isoforms [eIF(iso)4E] are mRNA 5’ cap-binding proteins which control cap-dependent translation initiation (Gingras et al., 1999). They are also the most extensively studied susceptibility genes (Lellis et al., 2002), and particular research attention has been paid to the relationship between *eIF4E/eIF(iso)4E* and potyvirus infection. For instance, mutation in *eIF4E/eIF(iso)4E* resulted in loss of susceptibility to potyviruses in multiple hosts, such as Arabidopsis, tomato, pepper, lettuce, pea, peanut, and sugarcane (Ruffel et al., 2002, 2005; Nicaise et al., 2003; Gao et al., 2004; Xu et al., 2017; Yang et al., 2021). In most cases, the interaction between eIF4E/eIF(iso)4E and the viral genome-linked proteins (VPgs) of potyviruses is required for virus infection (Léonard et al., 2000; Charron et al., 2008; Tavert-Roudet et al., 2017; Yang et al., 2021). Moreover, *eIF4E/eIF(iso)4E* was also found to mediate recessive resistance to other viruses. The recessive mutant *cum1* in Arabidopsis showed reduced accumulation of a cucumovirus, and *cum1* was found to encode an eIF4E protein (Yoshii et al., 2004). The recessive bymovirus resistance locus *rym4* in barley was proved to correspond to the *eIF4E* gene (Kanyuka et al., 2005). In melon, an *eIF4E* allele confers resistance to a carmovirus, depending on the inefficient interaction between the viral 3’ cap-independent translational enhancer and eIF4E (Nieto et al., 2006; Truniger et al., 2008). In addition, the mutation of tobacco *eIF(iso)4E* genes was found to reduce the susceptibility to an umbravirus (Udagawa et al., 2020).

Another less studied *eIF4E* family member, *novel cap-binding protein* (*nCBP*), has been identified in Arabidopsis showing 41.44 and 40.50% nucleotide sequence identity with *eIF4E* and *eIF(iso)4E*, respectively, (Ruud et al., 1998). The nCBP protein also supports the translation initiation of capped mRNA *in vitro* and *in vivo* (Bush et al., 2009). In addition, nCBP has been reported to be involved in the infection of different viruses in various crops. *ncbp-1/ncbp-2* mutants in cassava exhibited high resistance to cassava brown streak disease (Gomez et al., 2019), and the *ncbp* mutant in Arabidopsis displayed resistance to several viruses in *Alphaflexiviridae* and *Betaflexiviridae*, which encode triple gene block proteins (TGBps; Keima et al., 2017). These results indicate that *nCBP*, similar to *eIF4E/eIF(iso)4E*, has the potential to act as a recessive resistance gene, which is worthy of further investigation in other crops.

Potato virus S (PVS, *Carlavirus*), potato virus M (PVM, *Carlavirus*), and potato virus X (PVX, *Potexvirus*), which all encode TGBps, are important viruses posing great threats to potato production (Wang et al., 2011, 2016; Keima et al., 2017). In this study, we found that *StnCBP* knockdown compromised PVS accumulation but not PVM and PVX. Further analysis demonstrated that the interaction between StnCBP and PVS coat protein (CP) is crucial for the *StnCBP*-mediated recessive resistance to PVS, while StnCBP interacts with the CPs of both PVS and PVM. Moreover, *NbnCBP* knockdown significantly reduced PVX accumulation but not PVM, and NbnCBP also interacted with both their CPs in *N. benthamiana*. These results indicate that the interaction between nCBP and CP is necessary but not sufficient for facilitating the accumulation of TGB-encoding viruses, which improves the understanding of the underlying mechanism for *nCBP* to act as a susceptibility gene.

## Materials and methods

### Plant materials and virus isolates

Potato variety (Eshu 3) and three transgenic lines with *StnCBP* silencing were used in the study. The plants were maintained on MS medium (Murashige and Skoog, 1962) supplemented with 4% sucrose *in vitro* under a 16 h of light/8 h of dark photoperiod with 400–1,000 μmol photons m^–2^ s^–1^ light intensity at 20°C and were transplanted into pots (12 cm) containing premixed soil in the greenhouse located in Huazhong Agricultural University (HZAU) under a 12 h of light/dark photoperiod with 90 μmol photons m^–2^ s^–1^ light intensity at 18–22°C.

Three virus isolates used in this study, PVS-HB7, PVX-HB3, and PVM-HB36, were collected from a local potato virus survey and maintained in tobacco or potato host plants under greenhouse conditions at HZAU. The virus identity and purity were determined as described previously (Nie et al., 2012; Wang et al., 2016).

### BLAST search and cloning

A BLASTn search was performed using the *AtnCBP* (Accession number: AF028809) nucleotide sequence as a query against the *S. tuberosum* genome (Spud DB) and the *Solanaceae* Genomics Network (SGN) to retrieve *nCBP* homolog sequences in potato and *N. benthamiana*, respectively. *StnCBP* and *NbnCBP* were amplified with specific primers (Supplementary Table 1) from the potato cultivar Eshu 3 and *N. benthamiana*, respectively, using Phanta Super-Fidelity DNA polymerase (Vazyme, China). Then, the PCR amplicons were cloned into the pCE-Zero vector (Vazyme, China). Five colonies were randomly selected and sent for sequencing (Sangon Biotech, China). To analyze the phylogenetic relationships of eIF4E orthologues in *S. tuberosum, Arabidopsis thaliana, Solanum lycopersicum*, and *Nicotiana benthamiana*, we performed a BLASTp search against the NCBI and SGN databases using the amino acid sequences of potato *eIF4E* family members as queries. The phylogenetic tree was generated using MEGA5.2 with neighbor-joining tree and 1000 bootstraps. The nCBP orthologues in *Oryza sativa* and *Zea mays* were also obtained by a BLASTp against the NCBI database. Then, an amino acid sequence alignment of nCBP orthologues from the above species was performed by DNAMAN to analyze whether the nCBP orthologues are conserved across species.

### Generation of transgenic potato lines

Based on the design principles of RNAi fragments (Wesley et al., 2001; Reynolds et al., 2004), a fragment with 249 bp near the 5’ end of *StnCBP* gene was selected as the target fragment for RNA interference. The nucleotide sequence of the target fragment returned no other homologous genes except for *StnCBP* in a BLASTn search against the *S. tuberosum* genome. The fragment was recombined into the *Xho*I and *Xba*I sites of the pHellsGate8 vector. *Agrobacterium tumefaciens* (*A. tumefaciens*) strain GV3101 containing the recombined pHellsGate8 constructs was employed to transform the microtuber slices of Eshu 3 for transgenic lines as previously described (Zhou et al., 2019). Primers for constructing the RNAi vector are shown in Supplementary Table 1.

### Virus-induced gene silencing assay

A 270-bp region in the 5’-terminus of *NbnCBP* was selected as the virus-induced gene silencing (VIGS) target using SGN VIGS Tool^1^ (Fernandez-Pozo et al., 2015). Then, it was amplified and inserted into the *Eco*RI and *Bam*HI sites of pTRV2 vector (named pTRV2: NbnCBP), and the correct construction of the vector was confirmed by sequencing. The plasmids pTRV1, pTRV2, and pTRV2: NbnCBP were transformed into *A. tumefaciens* strain GV3101 by electroporation. The leaves of 4-week-old *N. benthamiana* plants were inoculated with a mixture of *Agrobacterium* cultures containing pTRV1 and pTRV2: NbnCBP (final OD600 = 0.5) to induce NbnCBP silencing; a mixture of *Agrobacterium* cultures containing pTRV1 and empty pTRV2 was used as a negative control (Control). Two weeks later, the upper leaves were collected to identify the silencing efficiency. Primers for constructing the VIGS vector are shown in Supplementary Table 1.

### Virus inoculation

Potato plants at the 4–6 leaf stage were mechanically inoculated with wild-type virus inocula (leaf extract: about 1 *g*/10 ml leaf tissue homogenized in 10 mM phosphate buffer with 32 mM sodium sulfite, pH 7.5) as described previously (Singh et al., 2003) or agro-infiltrated with virus infectious clones as described previously (Sun et al., 2017). Foliage symptoms were monitored daily post-inoculation until harvest. For resistance assays, the upper non-inoculated leaves were sampled at 10 and 15 days post-inoculation (dpi).

### Identification of virus accumulation

Total RNA of partially collected samples was extracted by Total RNApure Kit (ZOMANBIO, Beijing, China). The first-strand cDNA synthesis was performed by All-In-One 5X RT MasterMix (Applied Biological Materials, Vancouver, Canada). Then, the accumulation level of the viruses was detected through quantitative reverse-transcription polymerase chain reaction (qRT-PCR). The qRT-PCR analyses were carried out in the LightCycler480 Real-Time PCR system^2^ using 2X qPCR Real-Time Kit (Applied Biological Materials, Vancouver, Canada). The accumulation level analysis was calculated by the 2^–ΔΔ^ ^Cq^ method based on the internal reference gene ef1α (Accession number: AB061263) or *actin* (Accession number: XM_016658880; Nicot et al., 2005). All histograms were made with GraphPad Prism. Primers for qRT-PCR are shown in Supplementary Table 1.

Another part of the collected samples was analyzed by double-antibody sandwich enzyme-linked immunosorbent assay to detect the accumulation of viruses at the protein level using virus-specific polyclonal antibodies (Agdia, Elkhart, IN, United States) according to the manufacturer’s protocol as previously described (Wang et al., 2016). Five drops (∼100 μl) of leaf sap obtained by a tuber slicer (Elektrowerk, Hannover, Germany) were used as samples for the ELISA assay. The sap from virus-containing plants and virus-free plants was used as the positive controls and negative controls, respectively. After the samples were incubated with specific primary and secondary antibodies in 96-well ELISA plates, the chromogenic substrate pNPP was added and the absorbance value at 405 nm (A_405_) was measured using an ELx800 Universal Microplate Reader (Bio-Tek Instruments, Winooski, United States).

### Yeast-two-hybrid assay

StnCBP, NbnCBP, and AtnCBP were recombined between the *Bam*HI and *Eco*RI sites of the pGBKT7 vector, while the proteins of PVX and PVS were recombined between the *Bam*HI and *Eco*RI sites of the pGADT7 vector using SE cloning kit (Applied Biological Materials, Vancouver, Canada). Pairwise combinations for interaction analyses were co-transformed into yeast strain AH109 following BD Matchmaker Screening Kit. The interactions were identified through a medium that lacks tryptophan, leucine, adenine, and histidine and contains X-α-GAL (20 mg/L) as the interaction leads to blue plaques. Primers for constructing yeast-two-hybrid (Y2H) vectors are shown in Supplementary Table 2.

### Bimolecular fluorescent complimentary

Paired combinations for interaction analysis were inserted into nYFP and cYFP plasmids, respectively, through restriction endonuclease sites *Bam*HI and *Sal*I, and were introduced into *Agrobacterium* strain GV3101. Then, a mixture of *Agrobacterium* cultures containing the two recombinant plasmids (final OD600 = 0.2) was infiltrated into *N. benthamiana* leaves at the 4–6 leaf stage, and the fluorescence signal of YFP was observed at 48 h post-infiltration using a confocal laser scanning microscope (SP8, Leica, Wetzlar, Germany). Primers for constructing bimolecular fluorescent complimentary (BiFC) vectors are shown in Supplementary Table 3.

### Subcellular localization

The sequences coding for amino acids in protein (CDS) of *StnCBP* and PVS *CP* were amplified with specific primers and recombined into the *Bsp*1407I site of pK7WGF2 where fused behind eGFP for subcellular localization, and CDS of St*nCBP* was also inserted into the *Bsp*1407I site of pK7WGR2 where fused behind RFP for co-localization analysis. The vectors were electroporated into *Agrobacterium tumefaciens* strain GV3101 and agro-infiltrated the leaves of 4-week-old *N. benthamiana* as protocols previously described (Zhan et al., 2019). The fluorescence signal was observed at 48 h post-infiltration using a confocal laser scanning microscope (SP8, Leica, Wetzlar, Germany). Primers for constructing subcellular localization vectors are shown in Supplementary Table 3.

### Western blotting

Total proteins were extracted from leaves of *N. benthamiana* which expressed the GFP fused StnCBP and CP proteins using a protein extraction buffer [100 mM Tris–HCl (pH 8), 150 mM NaCl, 2 mM dithiothreitol, 5 mM EDTA, 10% glycerol, 2 mM phenylmethylsulfonyl fluoride, and 1% protease inhibitor tablets (A32955; Thermo Fisher Scientific)]. About 20 μl of 5× SDS-PAGE loading buffer with 5 μM β-mercaptoethanol was added to 80 μl of the extracted protein supernatant. The supernatant was heated to 95°C for 5 min to denature the protein, and then, these samples were loaded for Western blotting analysis. Probes with specific anti-GFP antibodies at 1:5,000 dilution (MBL, Japan) were used for Western blotting as protocols previously described (Cheng et al., 2021; Wang et al., 2021).

## Results

### *StnCBP* was an *eIF4E* gene belonging to the nCBP subgroup in potato

**FIGURE 1:**
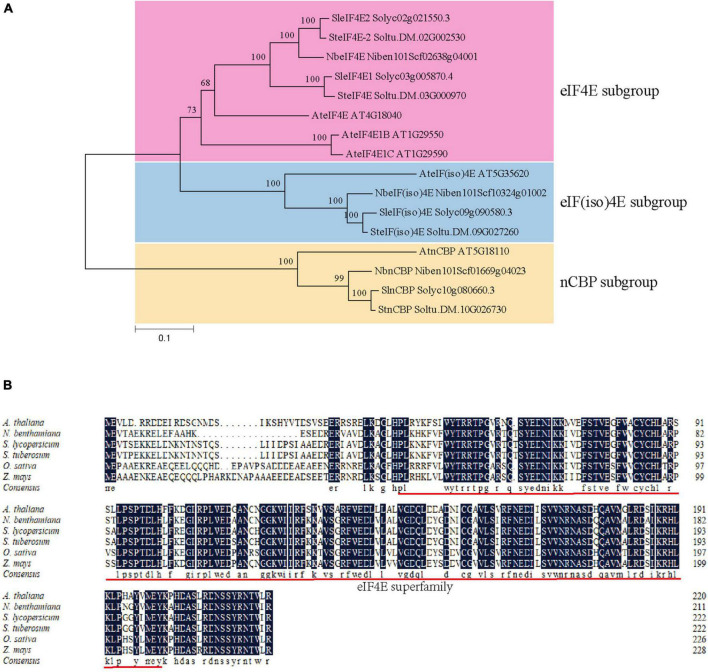
Phylogenetic analysis of *eIF4E* genes in potato, Arabidopsis, tobacco, and tomato **(A)**, and an amino acid sequence alignment of ncbp proteins from various species **(B)**. The prefix of accession numbers with “Soltu.DM” represents proteins from *S. tuberosum*; the prefix of accession numbers with “AT” represents proteins from *A. thaliana*; the prefix of accession numbers with “Solyc” represents proteins from *S. lycopersicum*; the prefix of accession numbers with “Niben101Scf” represents proteins from *N. benthamiana.* The eIF4E subgroup, eIF(iso)4E subgroup, and nCBP subgroup are distinguished by pink, blue, and yellow regions, respectively. The phylogenetic tree was generated with neighbor-joining tree and 1000 bootstraps. The red underline represents the eIF4E superfamily domain.

A BLASTn search against *the S. tuberosum* genome in the Spud DB database^3^ was performed using the nucleotide sequence of *nCBP* in *A. thaliana* (*AtnCBP*, Accession number: AT5G18110) as a query to identify *nCBP* orthologues in potato. As a result, a unique *nCBP* gene (named as *StnCBP*; Accession number: Soltu.DM.10G026730) was identified, which exhibited 59.6 and 70.0% homology to *AtnCBP* in nucleic acid and amino acid sequence, respectively. Potato has four members of the *eIF4E* family, including *SteIF4E* (Accession number: Soltu.DM.03G000970), *SteIF4E-2* (Accession number: Soltu.DM.02G002530), *SteIF(iso)4E* (Accession number: Soltu.DM.09G027260), and *StnCBP*. Then, we performed a BLASTp search against the NCBI and SGN databases using the amino acid sequences of potato *eIF4E* family members as queries. As a result, 12 eIF4E proteins were obtained from *A. thaliana* and two other *Solanaceae* crops (*N. benthamiana* and *S. lycopersicum*). The phylogenetic tree analysis of eIF4E proteins from the above species indicated that StnCBP belongs to the nCBP subgroup (Figure 1A). In addition, an amino acid sequence alignment of nCBP proteins from various species (including the above species, and *O. sativa*, and *Z. mays*) revealed that they contain an eIF4E superfamily domain and share a high homology of 82.32%, indicating that *nCBP* is highly conserved among different species and may have similar functions (Figure 1B).

### *StnCBP* knockdown compromised potato virus S accumulation

**FIGURE 2:**
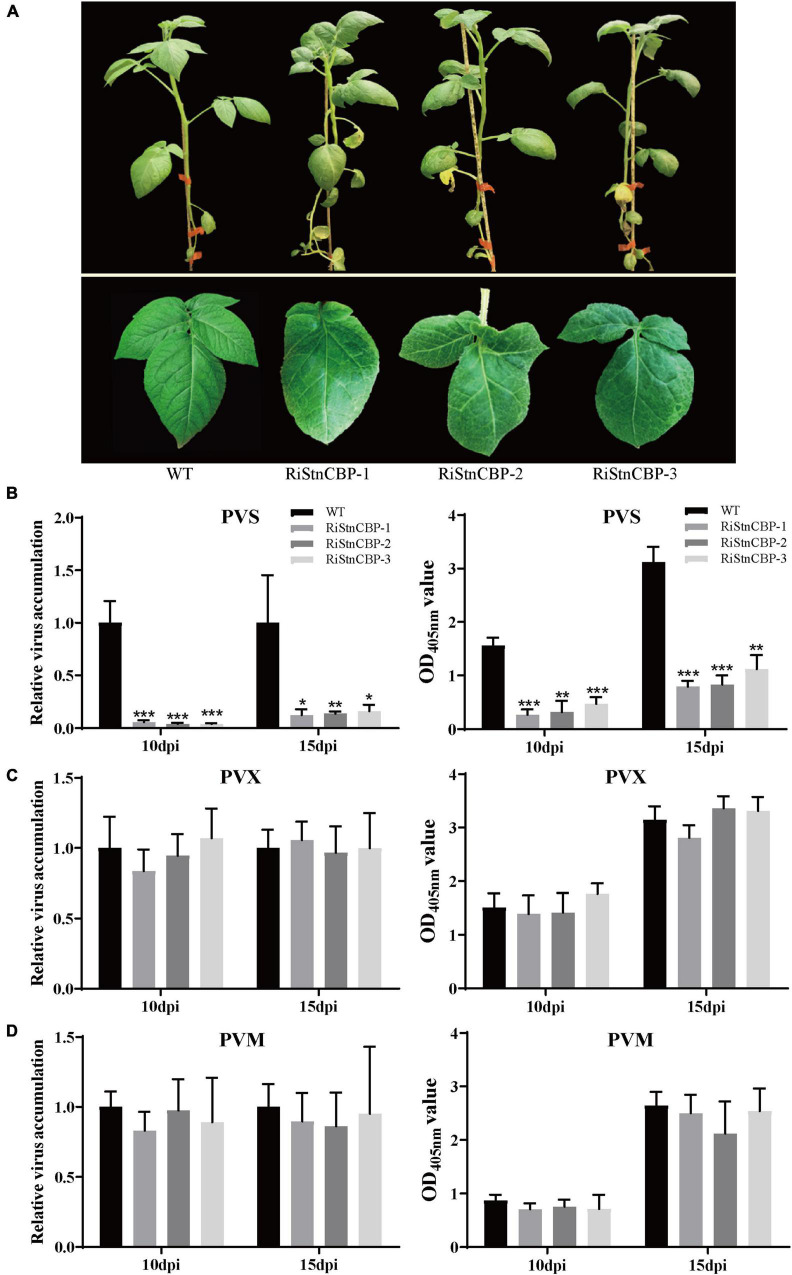
Morphology and virus resistance identification of the RiStnCBP transgenic lines. **(A)** Morphology of RiStnCBP transgenic lines under field conditions. **(B–D)** Virus resistance identification in RiStnCBP transgenic lines against PVS **(B)**, PVM **(C)**, and PVX **(D)**, respectively. The virus accumulation was determined using qRT-PCR (left) and ELISA (right). Error bars indicate ± SD of three replicates (*n* = 3). Three independent experiments were performed with similar results. *p* < 0.05*, *p* < 0.01**, and *p* < 0.001*** (Student’s *t*-test).

To determine whether StnCBP is also involved in the infection of important potato viruses encoding TGBps, RNA interference was performed to silence *StnCBP* expression. Several RNAi transgenic lines were obtained (the interference efficiency is shown in Supplementary Figure 1). The three transgenic lines with the highest interference efficiency (RiStnCBP-1, RiStnCBP-2, and RiStnCBP-3) were selected for further experiments. As shown in Figure 2A, *StnCBP* knockdown resulted in deformed compound leaves, indicating that *StnCBP* plays an important role in leaf development, but does not affect plant growth, which may be due to functional redundancy among eIF4Es. The expression of other members of the *SteIF4E* family was also examined in the RiStnCBP lines, and the results showed that the expression of *SteIF4E*, *SteIF4E-2*, and *SteIF(iso)4E* was not affected by the transgenic events (Supplementary Figure 2). Then, the RiStnCBP lines were mechanically inoculated with three TGB-encoding viruses, PVS, PVM, and PVX. No apparent symptom was observed after inoculation. The upper non-inoculated leaves were collected from control plants (WT) and transgenic lines at 10 and 15 dpi. Subsequently, the accumulation of these viruses was determined by qRT-PCR and ELISA assays. PVS accumulation was drastically reduced in the transgenic lines compared with that in WT plants (Figure 2B). In contrast, the accumulation of PVM and PVX in the RiStnCBP lines was at a similar level to that in WT plants (Figures 2C,D). These results indicated that *StnCBP* knockdown suppresses PVS accumulation but not PVM and PVX, which is the first report that a member of the *eIF4E* family is involved in PVS infection.

### StnCBP interacted with potato virus S coat protein

**FIGURE 3:**
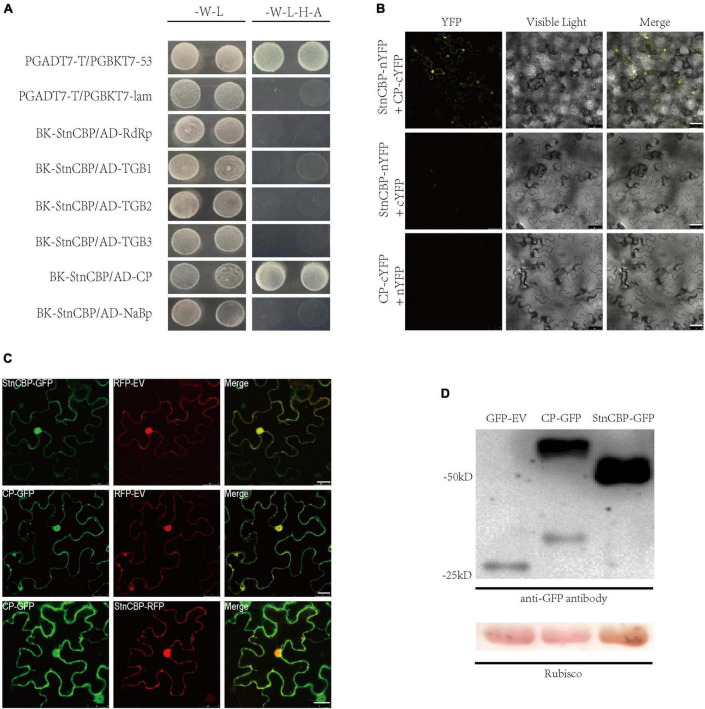
StnCBP interacted with PVS CP in the nucleus and cytoplasm. **(A)** Interaction between StnCBP and proteins of PVS in Y2H assays. -W-L represents a medium lacking tryptophan and leucine, while -W-L-H-A represents a medium lacking tryptophan, leucine, histidine, and adenine. Paired combinations PGADT7-T/PGBKT7-53 and PGADT7-T/PGBKT7-lam represent positive and negative controls, respectively. **(B)** Interaction between StnCBP and PVS CP in BiFC assays. Merge means the overlay of YFP and visible light on single confocal planes. Scale bar: 50 μm. **(C)** Subcellular localization and co-localization of StnCBP and PVS CP. Merge means the overlay of GFP and RFP on single confocal planes. Scale bar: 20 μm. **(D)** Identification of full-length proteins of StnCBP and PVS CP successfully expressed with GFP in the Western blotting. Total proteins were immunoprecipitated with anti-GFP antibody, and Rubisco was stained with Ponceau S as a loading control.

Many susceptibility factors have been proved to function by directly interacting with viral proteins (Roudet-Tavert et al., 2007; Hwang et al., 2015; Zhang et al., 2015). To determine whether StnCBP directly interacts with any protein of PVS, we tested the interaction between StnCBP and six proteins of PVS (including RdRp, TGB1, TGB2, TGB3, CP, and NaBp proteins) in Y2H assays (Figure 3A). The results revealed that StnCBP interacted with the CP of PVS, which was further demonstrated *via* BiFC assays in *N. benthamiana* (Figure 3B). Moreover, the results of BiFC demonstrated that StnCBP and PVS CP interacted in the nucleus and cytoplasm. Therefore, we further observed their subcellular localization and co-localization. StnCBP and PVS CP tagged with a GFP at the C-terminus were co-expressed with an RFP-empty vector (RFP-EV) in the leaves of *N. benthamiana* to observe their subcellular localization. As a result, the GFP fluorescence of StnCBP and PVS CP was both localized in the nucleus and cytoplasm (Figure 3C). To validate the successful expression of full-length StnCBP and PVS CP with GFP, Western blotting was performed to identify the protein size expressed by the vectors GFP-EV, StnCBP-GFP, and CP-GFP. They were predicted to encode proteins with sizes of 25.6, 52.5, and 59.4 kD, respectively, and the Western blotting results were consistent with expectation (Figure 3D), indicating the correct expression of proteins and accurate subcellular localization. Moreover, StnCBP-RFP and CP-GFP were found to be co-localized in the nucleus and cytoplasm (Figure 3C). These results indicated that StnCBP and PVS CP interact with each other and may function in the nucleus and cytoplasm.

We also tested the interaction between StnCBP and the proteins of PVX or PVM (Supplementary Figure 3). No interaction was observed between StnCBP and any PVX protein. Surprisingly, StnCBP also interacted with PVM CP, but *StnCBP* knockdown did not affect PVM infection. Therefore, we further determined whether PVS CP is critical for *StnCBP*-mediated recessive resistance to PVS.

### Potato virus S coat protein played a vital role in *StnCBP*-mediated recessive resistance to potato virus S

**FIGURE 4:**
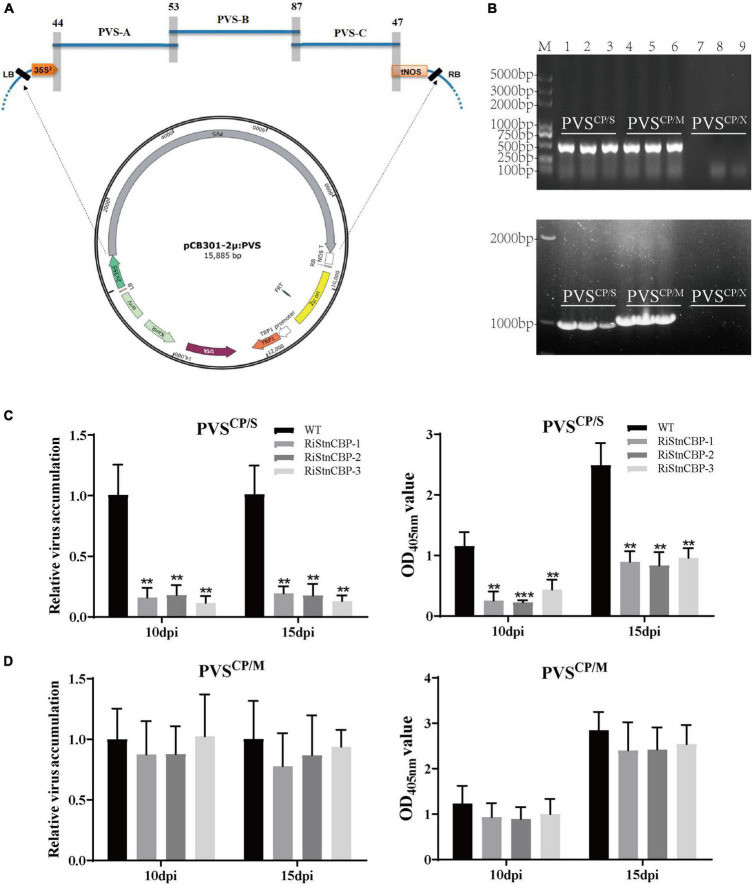
PVS CP played a vital role in the *StnCBP*-mediated recessive resistance to PVS. **(A)** Schematic illustration of the construction of PVS infectious clone. PVS-A, PVS-B, and PVS-C represent the fragments from PVS for the assembly. The gray shadow bars indicate the overlapping regions of adjacent fragments, with the number of overlapping nucleotides. **(B)** Electrophoretic separation of PCR products amplified with detection primers (above) and CP-flanking primers (below) from plants agroinoculated with PVS^CP/S^ (1–3), PVS^CP/M^ (4–6), and PVS^CP/X^ (7–9). M: Trans2K^®^ Plus DNA Marker. The upper non-inoculated leaves were collected 14 days after inoculation. **(C,D)** The accumulation of PVS^CP/S^ **(C)** and PVS^CP/M^ **(D)** infectious clones in the RiStnCBP and control plants (WT). The virus accumulation was determined using qRT-PCR (left) and ELISA (right). Error bars indicate ± SD of three replicates (*n* = 3). Three independent experiments were performed with similar results. *p* < 0.01** and *p* < 0.001*** (Student’s *t*-test).

Construction of plant RNA virus infectious clones has become a powerful tool to study virus molecular biology (Bao et al., 2020). Therefore, we constructed a PVS infectious clone to test whether PVS CP plays an important role in *StnCBP*-mediated recessive resistance to PVS. According to the previously described method (Sun et al., 2017), the PVS genome was divided into three partially overlapping cDNA fragments (PVS-A, PVS-B, and PVS-C), followed by amplification using specific primers (Supplementary Table 4) and homologous recombination assembly in the yeast with an expression vector pCB301-2μ-HDV to construct the full-length clone of PVS (named as pCB301-2μ: PVS; Figure 4A). Then, the CP in PVS infectious clone was replaced by the CP of PVM or PVX, and the infectious clones were designated as PVS^CP/S^, PVS^CP/M^, and PVS^CP/X^, respectively, (Supplementary Figure 4).

To confirm the infectivity, we inoculated the WT plants with the three infectious clones by *Agrobacterium* infiltration and detected the presence of the virus in the upper non-inoculated leaves by PCR assays at 14 dpi, with two pairs of primers. The first pair of primers (named as detection primers) were designed on the *TGB1* region of PVS for general detection. The other pair of primers (named as CP-flanking primers) were designed based on the sequences flanking the *CP* region, to confirm the substitution of viral CP according to the different sizes of CPs from PVS, PVM, and PVX (Supplementary Figure 5). As a result, PVS^CP/S^ and PVS^CP/M^ infectious clones successfully infected the plants as indicated by the amplification of PCR products with expected sizes (Figure 4B). In addition, the nucleotide lengths of *CP* genes of PVS and PVM were 885 bp and 915 bp, respectively. As expected, the PCR product amplified by CP-flanking primers from the PVS*^CP/M^* inoculated plants was slightly larger than that from PVS*^CP/S^* inoculated plants (Figure 4B), indicating correct substitution of the CP. Surprisingly, PVS^CP/X^ showed no infection ability since no PCR product was amplified (Figure 4B). A plausible explanation is that both PVS and PVM belong to the *Carlavirus* genus with mutually compatible CPs, while PVX belongs to the *Potexvirus* genus with an incompatible CP.

Furthermore, the RiStnCBP lines were inoculated with the PVS^CP/S^ and PVS^CP/M^ infectious clones by *Agrobacterium* infiltration. The upper non-inoculated leaves were collected at 10 and 15 dpi to determine the virus accumulation using qRT-PCR and ELISA assays. The RiStnCBP lines also had significantly lower accumulation of PVS^CP/S^ infectious clone than the control plants (Figure 4C). In contrast, the accumulation of PVS^CP/M^ infectious clone showed no significant difference between the RiStnCBP and control plants (Figure 4D). These results indicated that PVS CP is vital for *StnCBP*-mediated recessive resistance to PVS. However, the interaction between StnCBP and CP may be necessary but not sufficient for the *StnCBP*-mediated recessive resistance to TGB-encoding viruses.

### The *nCBP* orthologue in *N. benthamiana* was involved in potato virus X infection

**FIGURE 5:**
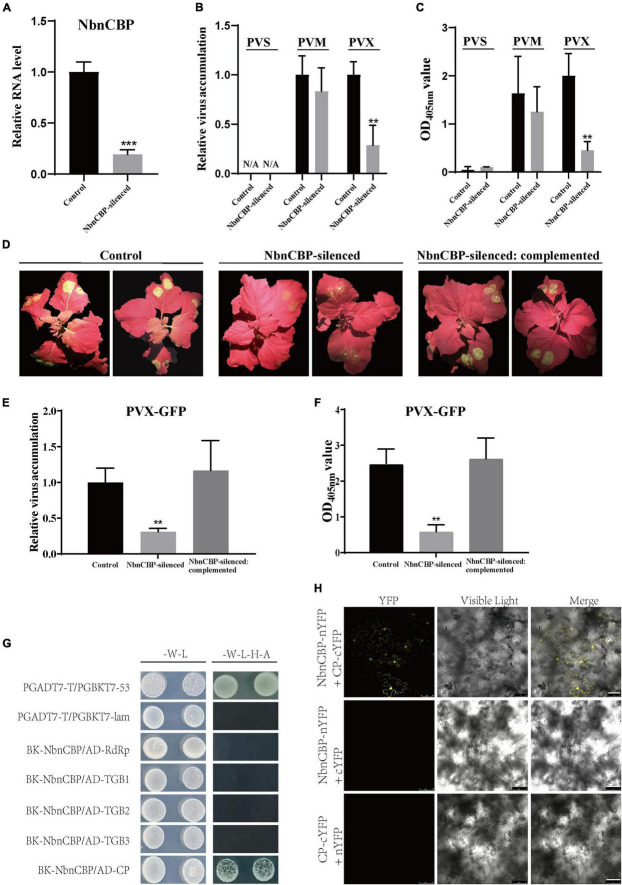
Antiviral identification of *NbnCBP* against PVX, and interaction analyses between NbnCBP and proteins of PVX. **(A)** The silencing efficiency of *NbnCBP* in *N. benthamiana* by VIGS. The gene expression was determined using qRT-PCR. Error bars indicate ± SD of five replicates (*n* = 5). *p* < 0.001*** (Student’s *t*-test). **(B,C)** The determination of PVM and PVX accumulation in the control and *NbnCBP*-silenced plants by RT-qPCR **(B)** and ELISA **(C)**. Error bars indicate ± SD of three replicates (*n* = 3). Three independent experiments were performed with similar results. *p* < 0.01** (Student’s *t*-test). **(D)** Green fluorescence emission in PVX-GFP inoculated leaves of the control, *NbnCBP*-silenced, and *NbnCBP*-silenced: complemented plants. The photographs were taken at 8 dpi. **(E,F)**. The determination of PVX-GFP accumulation in inoculated leaves of the control, *NbnCBP*-silenced and *NbnCBP*-silenced: complemented plants by RT-qPCR **(E)** and ELISA **(F)**. Error bars indicate ± SD of three replicates (*n* = 3). Three independent experiments were performed with similar results. *p* < 0.01** (Student’s *t*-test). **(G)** Interaction analysis between NbnCBP and the proteins of PVX in Y2H. -W-L represents a medium lacking tryptophan and leucine, while -W-L-H-A represents a medium lacking tryptophan, leucine, histidine, and adenine. Paired combinations PGADT7-T/PGBKT7-53 and PGADT7-T/PGBKT7-lam represent positive and negative controls, respectively. **(H)** Interaction analysis between NbnCBP and PVX CP in BiFC assay. nYFP and cYFP represent their empty vectors. Merge means the overlay of YFP and visible light on single confocal planes. Scale bar: 50 μm.

The function of *nCBP* gene in the infection of TGB-encoding viruses was first reported in Arabidopsis and then in potato in this study. However, it remains unknown whether the function of *nCBP* homolog is also conserved in *N. benthamiana*, which also belongs to the *Solanaceae* as potato. Therefore, the TRV-mediated VIGS assay was employed to silence the *nCBP* homolog in *N. benthamiana* (*NbnCBP*; accession number: Niben101Scf01669g04023.1). The silencing efficiency of *NbnCBP* by VIGS reached 80% (Figure 5A). The *NbnCBP*-silenced plants were then mechanically inoculated with PVS, PVM, and PVX. At 6 dpi, the upper non-inoculated leaves were collected to determine the virus accumulation by qRT-PCR and ELISA assays. Surprisingly, the accumulation of PVX was significantly reduced, while that of PVM was not affected in the *NbnCBP*-silenced plants, which was inconsistent with the results obtained for potato (Figures 5B,C). In addition, *N. benthamiana* exhibited resistance to PVS as no virus was detected in both *NbnCBP*-silenced and control plants (Figures 5B,C). It was reported that many tobacco varieties could not be infected by PVS in mechanical inoculation (De Bokx, 1970; Brattey et al., 2002; Nie and Singh, 2013), indicating that *N. benthamiana* may have some unknown resistance against PVS like these tobaccos. Therefore, it remains unclear whether NbnCBP is involved in the infection of PVS.

To provide more intuitive results, the *NbnCBP*-silenced plants were inoculated with a PVX infectious clone tagged with a green fluorescent protein (hereafter referred to as PVX-GFP) by agroinfiltration. The GFP fluorescence at 8 dpi revealed that PVX-GFP accumulation drastically decreased in the inoculated leaves, and the systemic infection was significantly delayed (Figure 5D). Further qRT-PCR and ELISA assays demonstrated that both the PVX RNA and protein levels decreased in *NbnCBP*-silenced plants compared with those in the control (Figures 5E,F). Obviously, these results were consistent with those of GFP fluorescence emission, indicating that *NbnCBP* knockdown compromises PVX-GFP accumulation.

A transient complementation assay was performed to confirm that the decrease in the accumulation of PVX in *NbnCBP*-silenced plants was directly caused by loss of *NbnCBP*. The leaves of *NbnCBP*-silenced plants were agro-infiltrated with a mixture of *Agrobacterium* cultures carrying the PVX-GFP infectious clone and a full-length cDNA of *NbnCBP*. As a result, the GFP fluorescence of PVX-GFP was recovered in the inoculated leaves of *NbnCBP*-silenced: complemented plants. However, the systemic infection of PVX-GFP was still delayed (Figure 5D). In addition, the qRT-PCR and ELISA results also revealed the PVX-GFP accumulation in the inoculated leaves of *NbnCBP*-silenced: complemented plants was comparable to the control plants (Figures 5E,F). Thus, it could be speculated that *NbnCBP* is required for PVX infection in *N. benthamiana*.

Then, a Y2H assay showed that the CP was the only protein interacting with NbnCBP in the five proteins coded by PVX, which was similar to the relationship between StnCBP and PVS CP (Figure 5G), and the interaction between NbnCBP and CP of PVX was further confirmed by the BiFC assay (Figure 5H). Thus, PVX CP may also have a critical function in *NbnCBP*-mediated recessive resistance against PVX. In addition, we analyzed the interaction between NbnCBP and the proteins of PVM and PVS. Interestingly, like the case in potato, NbnCBP interacted with PVM CP, though *NbnCBP* knockdown did not affect PVM infection (Supplementary Figures 6A,B). NbnCBP also interacted with PVS CP (Supplementary Figures 6C,D), even if PVS did not infect *N. benthamiana.* This result implies that NbnCBP may also have the function to facilitate PVS accumulation, but this phenotype was masked by other possible anti-PVS mechanisms prevalent in tobacco.

In summary, our results indicated that the *nCBP* orthologues have conserved functions in potato and tobacco as in Arabidopsis in terms of recessive resistance against TGB-encoding viruses, and the interaction between nCBP and CP is necessary but not sufficient to determine the recessive resistance of *nCBP*.

## Discussion

Recessive resistance genes have become a very important resource in plant breeding for virus resistance (Truniger and Aranda, 2009). *eIF4Es* are the most extensively studied recessive resistance genes involved in the infection of various viruses (Hashimoto et al., 2016; Xu et al., 2017; Yang et al., 2021). This study for the first time reports that *StnCBP*, a member of the *eIF4E* family in potato, is involved in PVS infection as a recessive resistance gene. PVS has become an important virus in all potato-growing areas worldwide (Song et al., 2017). However, compared with potato virus Y resistance, potato resistance to PVS has been much less studied, except for the chromosomal localization of a dominant gene (*Ns*) against PVS (Marczewski et al., 2002). This study identified a recessive resistance gene (*StnCBP*) to PVS, which may provide a new strategy for developing potato varieties resistant to PVS.

Numerous studies of *eIF4Es* have suggested that their interactions with VPg proteins are responsible for the susceptibility to potyviruses. For instance, the mutation of *eIF4Es* that abolishes their interaction with VPg proteins confers resistance to potyviruses, and, in turn, some isolates of potyviruses can restore the interaction to lead to a resistance-breaking phenotype (Yeam et al., 2007; Charron et al., 2008; Truniger and Aranda, 2009). In this study, *StnCBP* knockdown compromised PVS accumulation but not PVX and PVM (Figure 2), and further assays demonstrated that StnCBP directly interacts with PVS CP both *in vitro* and *in vivo* (Figure 3). These results indicated that PVS CP may also be crucial for the function of *StnCBP* just like VPgs for *eIF4Es*. We further confirmed the speculation since the substitution of PVS CP by PVM CP recovered the virus accumulation in the RiStnCBP lines (Figure 4). Furthermore, our results also suggested that *NbnCBP* is required for PVX infection when it interacts with PVX CP (Figure 5), indicating that nCBP-CP interaction may be a common mode in *nCBP*-mediated recessive resistance against TGB-encoding viruses.

However, how nCBP facilitates TGB-encoding virus accumulation is still elusive. nCBP was found to be a component of the 5 ‘cap complex in Arabidopsis and supports the translation initiation of capped mRNA, like eIF4E/eIF(iso)4E (Ruud et al., 1998; Bush et al., 2009). The genomes of PVS, PVM, and PVX consist of five or six open reading frames, a 5’ cap and a 3’ poly(A) tail (Poke, 2008; Petrova et al., 2013). Studies on the PVX suggested that the 5’ cap structure of RNA and PVX CP plays crucial role for viral ribonucleoprotein (vRNP) assembly (Petrova et al., 2013, 2015). Therefore, the interaction between nCBP and viral CP may promote vRNP assembly, thereby regulating viral translation and facilitating the accumulation of TGB-encoding viruses.

Notably, the knockdown of *StnCBP* and *NbnCBP* did not affect PVM infection in *S. tuberosum* and *N. benthamiana*, respectively, whereas both StnCBP and NbnCBP interacted with PVM CP. One reasonable explanation is that knockdown instead of knockout was used to identify the function of *nCBP* homolog genes, which still produced a certain amount of transcripts. Thus, it is possible that the effect on PVM would only be observed in a knockout situation. In fact, the *ncbp* mutation in Arabidopsis significantly reduced the accumulation of PVM (Keima et al., 2017), and AtnCBP was found to interact with PVM CP in both the Y2H and BiFC assays (Supplementary Figures 7A,B), which supports this explanation. Further knockout analysis is needed to confirm the recessive resistance of the *nCBP* orthologues in potato and tobacco to these TGB-encoding viruses.

Another possible explanation is that the nCBP-CP interaction may be a necessary but not sufficient condition for *nCBP* to act as a susceptibility gene, and some other viral proteins may also be critical in the process. The TGBps are considered as conserved gene modules involved in the viral movement, which is similar to movement proteins (MPs) of some viruses (Morozov and Solovyev, 2003; Keima et al., 2017). TGB-encoding viruses are classified into two classes: the hordei-like class in which the CP is dispensable for cell-to-cell movement of viruses, and the potex-like class in which the CP is required for cell-to-cell movement (Forster et al., 1992; Solovyev et al., 1996; Wong et al., 1998; Lin et al., 2006). The potex-like class viruses have a TGB1 ranging from 24 to 26 kDa and a TGB3 of about 7 kDa, while hordei-like class viruses contain a TGB1 ranging from 39 to 63 kDa and a TGB3 of about 15 kDa (Morozov and Solovyev, 2003; Lauber et al., 2005). In addition, the hordei-like class viruses are multicomponent viruses with rigid rod-shaped virions, while the potex-like class viruses are monopartite viruses with filamentous virions (Lauber et al., 2005). Thus, the TGBps of PVS, PVM, and PVX can be classified into the potex-like class (Morozov and Solovyev, 2003), implying that their CPs may be required for TGBps to establish the cell-to-cell movement and systemic infection of viruses. In addition, a previous study has shown that *ncbp* mutation in Arabidopsis conferred resistance to serval potex-like viruses (Keima et al., 2017). Among these viruses, the cell-to-cell movement of plantago asiatica mosaic virus (*Potexvirus*) was significantly delayed, and the accumulation of TGB2 and TGB3 was reduced, which established the association between the function of AtnCBP and TGBps. In addition, we analyzed the interaction between AtnCBP and the proteins of PVX by Y2H assays. As a result, AtnCBP interacted with the TGB2, TGB3, and CP of PVX (Supplementary Figure 7C), indicating the association among nCBP, TGBps, and CP.

In summary, this study identified *StnCBP* as a susceptibility gene required for the accumulation of a TGB-encoding virus PVS by recognizing its CP in potato. Our findings revealed that the nCBP-CP interaction may also contribute to the function of *nCBP* orthologues for other TGB-encoding viruses. Moreover, the cassava nCBP was found to interact with the VPgs of two ipomoviruses, which do not encode TGBps (Gomez et al., 2019). It will be meaningful to further study whether *nCBP* is required for the infection of other types of viruses and the underlying mechanism for *nCBP* to facilitate the infection of TGB-encoding and non-TGB-encoding viruses.

## Data availability statement

The original contributions presented in this study are included in the article/Supplementary material, further inquiries can be directed to the corresponding authors.

## Author contributions

CH, BN, RC, and XH conceived and designed the experiments. RC, MY, FX, and TL performed the experiments. RC, ZT, and JC analyzed the data. RC, CH, and BN prepared the manuscript. CH, BN, and XH revised the manuscript. All authors contributed to the article and approved the submitted version.

## Footnotes

1
https://vigs.solgenomics.net/

2
https://diagnostics.roche.com

3
http://spuddb.uga.edu/

## References

[B1] Bao W. Yan T. Deng X. Wuriyanghan H. (2020). Synthesis of full-length cDNA infectious clones of Soybean mosaic virus and functional identification of a key amino acid in the silencing suppressor Hc-Pro. *Viruses* 12:886. 10.3390/v12080886 32823665 PMC7472419

[B2] Brattey C. Badge J. Burns R. Foster G. George E. Goodfellow H. (2002). Potato latent virus: a proposed new species in the genus Carlavirus. *Plant Pathol.* 51 495–505. 10.1046/j.1365-3059.2002.00729.x

[B3] Bush M. S. Hutchins A. P. Jones A. M. Naldrett M. J. Jarmolowski A. Lloyd C. W. (2009). Selective recruitment of proteins to 5’ cap complexes during the growth cycle in Arabidopsis. *Plant J.* 59 400–412. 10.1111/j.1365-313X.2009.03882.x 19453450

[B4] Charron C. Nicolaï M. Gallois J. L. Robaglia C. Moury B. Palloix A. (2008). Natural variation and functional analyses provide evidence for co-evolution between plant eIF4E and potyviral VPg. *Plant J.* 54 56–68. 10.1111/j.1365-313X.2008.03407.x 18182024

[B5] Cheng D. Zhou D. Wang Y. Wang B. He Q. Song B. (2021). Ralstonia solanacearum type III effector RipV2 encoding a novel E3 ubiquitin ligase (NEL) is required for full virulence by suppressing plant PAMP-triggered immunity. *Biochem. Biophys. Res. Commun.* 550 120–126. 10.1016/j.bbrc.2021.02.082 33691198

[B6] De Bokx J. (1970). Reactions of various plant species to inoculation with potato virus S. *Neth. J. Plant Pathol.* 76 70–78. 10.1007/BF01974435

[B7] Fernandez-Pozo N. Rosli H. G. Martin G. B. Mueller L. A. (2015). The SGN VIGS tool: user-friendly software to design virus-induced gene silencing (VIGS) constructs for functional genomics. *Mol. Plant* 8 486–488. 10.1016/j.molp.2014.11.024 25667001

[B8] Forster R. L. Beck D. L. Guilford P. J. Voot D. M. Van Dolleweerd C. J. Andersen M. T. (1992). The coat protein of white clover mosaic potexvirus has a role in facilitating cell-to-cell transport in plants. *Virology* 191 480–484. 10.1016/0042-6822(92)90215-B 1413520

[B9] Gao Z. Johansen E. Eyers S. Thomas C. L. Noel Ellis T. Maule A. J. (2004). The potyvirus recessive resistance gene, sbm1, identifies a novel role for translation initiation factor eIF4E in cell-to-cell trafficking. *Plant J.* 40 376–385. 10.1111/j.1365-313X.2004.02215.x 15469495

[B10] Garcia-Ruiz H. (2018). Susceptibility genes to plant viruses. *Viruses* 10:484. 10.3390/v10090484 30201857 PMC6164914

[B11] Gingras A. C. Raught B. Sonenberg N. (1999). eIF4 initiation factors: effectors of mRNA recruitment to ribosomes and regulators of translation. *Annu. Rev. Biochem.* 68 913–963. 10.1146/annurev.biochem.68.1.913 10872469

[B12] Gomez M. A. Lin Z. D. Moll T. Chauhan R. D. Hayden L. Renninger K. (2019). Simultaneous CRISPR/Cas9-mediated editing of cassava eIF4E isoforms nCBP-1 and nCBP-2 reduces cassava brown streak disease symptom severity and incidence. *Plant Biotechnol. J.* 17 421–434. 10.1111/pbi.12987 30019807 PMC6335076

[B13] Hardigan M. A. Laimbeer F. P. E. Newton L. Crisovan E. Hamilton J. P. Vaillancourt B. (2017). Genome diversity of tuber-bearing Solanum uncovers complex evolutionary history and targets of domestication in the cultivated potato. *Proc. Natl. Acad. Sci. U.S.A.* 114 E9999–E10008. 10.1073/pnas.1714380114 29087343 PMC5699086

[B14] Hashimoto M. Neriya Y. Yamaji Y. Namba S. (2016). Recessive resistance to plant viruses: potential resistance genes beyond translation initiation factors. *Front. Microbiol.* 7:1695. 10.3389/fmicb.2016.01695 27833593 PMC5080351

[B15] Hwang J. Lee S. Lee J. H. Kang W. H. Kang J. H. Kang M. Y. (2015). Plant translation elongation factor 1Bβ facilitates potato virus X (PVX) infection and interacts with PVX triple gene block protein 1. *PLoS One* 10:e0128014. 10.1371/journal.pone.0128014 26020533 PMC4447259

[B16] Kanyuka K. Druka A. Caldwell D. G. Tymon A. McCallum N. Waugh R. (2005). Evidence that the recessive bymovirus resistance locus rym4 in barley corresponds to the eukaryotic translation initiation factor 4E gene. *Mol. Plant Pathol.* 6 449–458. 10.1111/j.1364-3703.2005.00294.x 20565670

[B17] Keima T. Hagiwara-Komoda Y. Hashimoto M. Neriya Y. Koinuma H. Iwabuchi N. (2017). Deficiency of the eIF4E isoform nCBP limits the cell-to-cell movement of a plant virus encoding triple-gene-block proteins in Arabidopsis thaliana. *Sci. Rep.* 7:39678. 10.1038/srep39678 28059075 PMC5216350

[B18] Lauber E. Jonard G. Richards K. Guilley H. (2005). Nonregulated expression of TGBp3 of hordei-like viruses but not of potex-like viruses inhibits beet necrotic yellow vein virus cell-to-cell movement. *Arch. Virol.* 150 1459–1467. 10.1007/s00705-005-0516-y 15770352

[B19] Lellis A. D. Kasschau K. D. Whitham S. A. Carrington J. C. (2002). Loss-of-susceptibility mutants of Arabidopsis thaliana reveal an essential role for eIF (iso) 4E during potyvirus infection. *Curr. Biol.* 12 1046–1051. 10.1016/S0960-9822(02)00898-9 12123581

[B20] Leìonard S. Plante D. Wittmann S. Daigneault N. Fortin M. G. Laliberteì J. F. (2000). Complex formation between potyvirus VPg and translation eukaryotic initiation factor 4E correlates with virus infectivity. *J. Virol.* 74 7730–7737. 10.1128/jvi.74.17.7730-7737.2000 10933678 PMC112301

[B21] Lin M. K. Hu C. C. Lin N. S. Chang B. Y. Hsu Y. H. (2006). Movement of potexviruses requires species-specific interactions among the cognate triple gene block proteins, as revealed by a trans-complementation assay based on the bamboo mosaic virus satellite RNA-mediated expression system. *J. Gen. Virol.* 87 1357–1367. 10.1099/vir.0.81625-0 16603539

[B22] Mäkinen K. (2020). Plant susceptibility genes as a source for potyvirus resistance. *Ann. Appl. Biol.* 176 122–129. 10.1111/aab.12562

[B23] Marczewski W. Hennig J. Gebhardt C. (2002). The Potato virus S resistance gene Ns maps to potato chromosome VIII. *Theor. Appl. Genet.* 105 564–567. 10.1007/s00122-002-0976-3 12582505

[B24] Morozov S. Y. Solovyev A. G. (2003). Triple gene block: modular design of a multifunctional machine for plant virus movement. *J. Gen. Virol.* 84 1351–1366. 10.1099/vir.0.18922-0 12771402

[B25] Murashige T. Skoog F. (1962). A revised medium for rapid growth and bio assays with tobacco tissue cultures. *Physiol. Plantar.* 15 473–497. 10.1111/j.1399-3054.1962.tb08052.x

[B26] Nicaise V. German-Retana S. Sanjuán R. Dubrana M. P. Mazier M. Maisonneuve B. (2003). The eukaryotic translation initiation factor 4E controls lettuce susceptibility to the potyvirus Lettuce mosaic virus. *Plant Physiol.* 132 1272–1282. 10.1104/pp.102.017855 12857809 PMC167067

[B27] Nicot N. Hausman J. F. Hoffmann L. Evers D. (2005). Housekeeping gene selection for real-time RT-PCR normalization in potato during biotic and abiotic stress. *J. Exp. Bot.* 56 2907–2914. 10.1093/jxb/eri285 16188960

[B28] Nie B. Singh M. Murphy A. Sullivan A. Xie C. Nie X. (2012). Response of potato cultivars to five isolates belonging to four strains of Potato virus Y. *Plant Dis.* 96 1422–1429. 10.1094/PDIS-01-12-0018-RE 30727313

[B29] Nie X. Singh M. (2013). Response of potato, tobacco and Physalis floridana plants to mixed infection with PVX, PVYNTN and PVY strains. *Can. J. Plant Pathol.* 35 390–401. 10.1080/07060661.2013.812581

[B30] Nieto C. Morales M. Orjeda G. Clepet C. Monfort A. Sturbois B. (2006). An eIF4E allele confers resistance to an uncapped and non-polyadenylated RNA virus in melon. *Plant J.* 48 452–462. 10.1111/j.1365-313X.2006.02885.x 17026540

[B31] Petrova E. K. Nikitin N. A. Protopopova A. D. Arkhipenko M. V. Yaminsky I. V. Karpova O. V. (2013). The role of the 5’-cap structure in viral ribonucleoproteins assembly from potato virus X coat protein and RNAs. *Biochimie* 95 2415–2422. 10.1016/j.biochi.2013.09.004 24036171

[B32] Petrova E. K. Nikitin N. A. Trifonova E. A. Protopopova A. D. Karpova O. V. Atabekov J. G. (2015). The 5 ’-proximal region of Potato virus X RNA involves the potential cap-dependent “conformational element” for encapsidation. *Biochimie* 115 116–119. 10.1016/j.biochi.2015.05.012 26006294

[B33] Poke F. S. (2008). Hop mosaic virus: complete nucleotide sequence and relationship to other carlaviruses. *Arch. Virol.* 153 1615–1619. 10.1007/s00705-008-0157-z 18607673

[B34] Rashid M. O. Li J. H. Liu Q. Wang Y. Han C. G. (2021). Molecular detection and identification of eight potato viruses in Gansu province of China. *Curr. Plant Biol.* 25:100184. 10.1016/j.cpb.2020.100184

[B35] Reynolds A. Leake D. Boese Q. Scaringe S. Marshall W. S. Khvorova A. (2004). Rational siRNA design for RNA interference. *Nature Biotechnol.* 22 326–330. 10.1038/nbt936 14758366

[B36] Roudet-Tavert G. Michon T. Walter J. Delaunay T. Redondo E. Le Gall O. (2007). Central domain of a potyvirus VPg is involved in the interaction with the host translation initiation factor eIF4E and the viral protein HcPro. *J. Gen. Virol.* 88 1029–1033. 10.1099/vir.0.82501-0 17325377

[B37] Ruffel S. Dussault M. H. Palloix A. Moury B. Bendahmane A. Robaglia C. (2002). A natural recessive resistance gene against potato virus Y in pepper corresponds to the eukaryotic initiation factor 4E (eIF4E). *Plant J.* 32 1067–1075. 10.1046/j.1365-313X.2002.01499.x 12492847

[B38] Ruffel S. Gallois J. L. Lesage M. Caranta C. (2005). The recessive potyvirus resistance gene pot-1 is the tomato orthologue of the pepper pvr2-eIF4E gene. *Mol. Genet. Genomics* 274 346–353. 10.1007/s00438-005-0003-x 15971038

[B39] Ruud K. A. Kuhlow C. Goss D. J. Browning K. S. (1998). Identification and characterization of a novel cap-binding protein from *Arabidopsis thaliana*. *J. Biol. Chem.* 273 10325–10330. 10.1074/jbc.273.17.10325 9553087

[B40] Singh R. P. McLaren D. L. Nie X. Singh M. (2003). Possible escape of a recombinant isolate of Potato virus Y by serological indexing and methods of its detection. *Plant Dis.* 87 679–685. 10.1094/pdis.2003.87.6.679 30812860

[B41] Solovyev A. Savenkov E. Agranovsky A. Morozov S. Y. (1996). Comparisons of the genomiccis-elements and coding regions in RNAβ components of the hordeiviruses Barley stripe mosaic virus, Lychnis ringspot virus, and Poa semilatent virus. *Virology* 219 9–18. 10.1006/viro.1996.0217 8623558

[B42] Song G. Wu J. Y. Xie Y. Liu Y. Qian Y. J. Zhou X. P. (2017). Monoclonal antibody-based serological assays for detection of Potato virus S in potato plants. *J. Zhejiang Univ. Sci. B* 18 1075–1082. 10.1631/jzus.B1600561 29204987 PMC5742290

[B43] Sun K. Zhao D. Liu Y. Huang C. Zhang W. Li Z. (2017). Rapid construction of complex plant RNA virus infectious cDNA clones for agroinfection using a yeast-*E. coli*-Agrobacterium shuttle vector. *Viruses* 9:332. 10.3390/v9110332 29112135 PMC5707539

[B44] Tavert-Roudet G. Anne A. Barra A. Chovin A. Demaille C. Michon T. (2017). The potyvirus particle recruits the plant translation initiation factor eIF4E by means of the VPg covalently linked to the viral RNA. *Mol. Plant Microbe Interact.* 30 754–762. 10.1094/MPMI-04-17-0091-R 28609214

[B45] Truniger V. Aranda M. A. (2009). Recessive resistance to plant viruses. *Adv. Virus Res.* 75 119–231. 10.1016/s0065-3527(09)07504-6 20109665

[B46] Truniger V. Nieto C. González-Ibeas D. Aranda M. (2008). Mechanism of plant eIF4E-mediated resistance against a Carmovirus (*Tombusviridae*): cap-independent translation of a viral RNA controlled in cis by an (a) virulence determinant. *Plant J.* 56 716–727. 10.1111/j.1365-313X.2008.03630.x 18643998

[B47] Udagawa H. Koga K. Shinjo A. Kitashiba H. Takakura Y. (2020). Reduced susceptibility to a tobacco bushy top virus Malawi isolate by loss of function in host eIF(iso)4E genes. *Breed. Sci.* 19135. 10.1270/jsbbs.19135 32714053 PMC7372031

[B48] van der Waals J. E. Krüger K. (2020). Emerging potato pathogens affecting food security in southern Africa: Recent research. *S. Afr. J. Sci.* 116 30–36. 10.17159/sajs.2020/8055

[B49] Wang B. He T. Zheng X. Song B. Chen H. (2021). Proteomic analysis of potato responding to the invasion of Ralstonia solanacearum UW551 and its type III secretion system mutant. *Mol. Plant Microbe Interact.* 34 337–350. 10.1094/mpmi-06-20-0144-r 33332146

[B50] Wang B. Ma Y. Zhang Z. Wu Z. Wu Y. Wang Q. (2011). Potato viruses in China. *Crop Prot.* 30 1117–1123. 10.1016/j.cropro.2011.04.001

[B51] Wang J. Meng F. Chen R. Liu J. Nie X. Nie B. (2016). RT-PCR differentiation, molecular and pathological characterization of Andean and ordinary strains of Potato virus S in potatoes in China. *Plant Dis.* 100 1580–1585. 10.1094/pdis-11-15-1257-re 30686236

[B52] Wesley S. V. Helliwell C. A. Smith N. A. Wang M. B. Rouse D. T. Liu Q. (2001). Construct design for efficient, effective and high-throughput gene silencing in plants. *Plant J.* 27 581–590. 10.1046/j.1365-313X.2001.01105.x 11576441

[B53] Wong S. M. Lee K. C. Yu H. H. Leong W. F. (1998). Phylogenetic analysis of triple gene block viruses based on the TGB1 homolog gene indicates a convergent evolution. *Virus Genes* 16 295–302. 10.1023/a:1008034807216 9654683

[B54] Xu M. Xie H. Wu J. Xie L. Yang J. Chi Y. (2017). Translation initiation factor eIF4E and eIFiso4E are both required for peanut stripe virus infection in peanut (*Arachis hypogaea* L.). *Front. Microbiol.* 8:338. 10.3389/fmicb.2017.00338 28344571 PMC5344889

[B55] Yang Z. Dong M. Cheng G. Liu S. Zhang H. Shang H. (2021). Selective interaction of sugarcane eIF4E with VPgs from sugarcane mosaic pathogens. *Viruses Basel* 13:518. 10.3390/v13030518 33809985 PMC8005120

[B56] Yeam I. Cavatorta J. R. Ripoll D. R. Kang B. C. Jahn M. M. (2007). Functional dissection of naturally occurring amino acid substitutions in eIF4E that confers recessive potyvirus resistance in plants. *Plant Cell* 19 2913–2928. 10.1105/tpc.107.050997 17890375 PMC2048695

[B57] Yoshii M. Nishikiori M. Tomita K. Yoshioka N. Kozuka R. Naito S. (2004). The Arabidopsis Cucumovirus multiplication 1 and 2 loci encode tip translation initiation factors 4E and 4G. *J. Virol.* 78 6102–6111. 10.1128/jvi.78.12.6102-6111.2004 15163703 PMC416505

[B58] Zhan X. Zhang F. Zhong Z. Chen R. Wang Y. Chang L. (2019). Generation of virus-resistant potato plants by RNA genome targeting. *Plant Biotechnol. J.* 17 1814–1822. 10.1111/pbi.13102 30803101 PMC6686122

[B59] Zhang L. Chen H. Brandizzi F. Verchot J. Wang A. (2015). The UPR branch IRE1-bZIP60 in plants plays an essential role in viral infection and is complementary to the only UPR pathway in Yeast. *PloS Genetics* 11:1005164. 10.1371/journal.pgen.1005164 25875739 PMC4398384

[B60] Zhou T. Song B. Liu T. Shen Y. Dong L. Jing S. (2019). Phytochrome F plays critical roles in potato photoperiodic tuberization. *Plant J.* 98 42–54. 10.1111/tpj.14198 30552774

