# Supplementary material for: Eukaryotic translation initiation factor 4E family member nCBP facilitates the accumulation of TGB-encoding viruses by recognizing the viral coat protein in potato and tobacco
Source: Front Plant Sci. 2022 Aug 8;13:946873. doi: 10.3389/fpls.2022.946873 (PMC9393630; doi:10.3389/fpls.2022.946873)
Supplement: Supplementary file 1 [file Data_Sheet_1.DOCX]

**Supplementary Material**

**Supplementary Table 1. Primers for gene cloning, constructing RNAi and VIGS vectors, and qRT-PCR.**

| **Primer name** | **Sequence (5'-3')** | | **Note** |
| --- | --- | --- | --- |
| StnCBP-F | | ATGGAAGTGACACCGGAGAAGA | to amplify full-length cDNA of *StnCBP* |
| StnCBP-R | | TTATGATCTTATCCAAGTATTGCGGTAAG |  |
| NbnCBP-F | | ATGGAAGTGACAGCTGAGAAGAG | to amplify full-length cDNA of *NbnCBP* |
| NbnCBP-R | | TTATCCTCTTATCCAAGTATTGCGGTATG |  |
| StnCBP-XhoIF | | tttggagaggacacgctcgagCTCAATAGCCGCCGAGGATC | to construct RNAi vector of *StnCBP* |
| StnCBP-XhoIR | | tggggtaccgaattcctcgagCCCTCTTTGAAAAGATGCAAATCAG |  |
| StnCBP-XhoIF | | tcattaaagcaggactctagaCTCAATAGCCGCCGAGGATC | to construct RNAi vector of *StnCBP* |
| StnCBP-XbaIR | | gataagcttggatcctctagaCCCTCTTTGAAAAGATGCAAATCAG |  |
| NbnCBP-VF | | tctgtgagtaaggttaccgaattc ATGGAAGTGACAGCTGAGAAGAG | to construct pTRV2:NbnCBP for VIGS |
| NbnCBP-VR | | acgcgtgagctcggtaccggatcc ATCAGTTGGGCTTGGCAATG |  |
| EF1α-F | | ATTGGAAACGGATATGCTCCA | reference gene for real time PCR |
| EF1α-R | | TCCTTACCTGAACGCCTGTCA |  |
| Actin-F | | CCTGTGGACAATGGATGGAC | reference gene for real time PCR |
| Actin-R | | ATCACGGCTTTGGCTCCTAG |  |
| StnCBP-QF | | TCGTAATGCGTCAGATCAGCAGG | primers for real time PCR |
| StnCBP-QR | | GCGCAAGGAAGCATCATGGG |  |
| SteIF4E-QF | | GGAGCAGTCGTTAGTGTCCG | primers for real time PCR |
| SteIF4E-QR | | GCACTTCTGTCGAGCCTCTT |  |
| SteIF4E2-QF | | TACCTGCTGGCTGTATACGC | primers for real time PCR |
| SteIF4E2-QR | | GCATTCCTGGTCCACAGAGC |  |
| SteIF(iso)4E-QF | | AAGCAGCTCAGATGGGCATT | primers for real time PCR |
| SteIF(iso)4E-QR | | TCGGCTCTTAGCTGACCTCT |  |
| NbnCBP-QF | | CGTCCATTATGGGAGGATGC | primers for real time PCR |
| NbnCBP-QR | | CTACCAAAGCCAGAACCAGGTC |  |
| PVS-QF | | GTTCCCACTGAGCATGTTGC | primers for real time PCR |
| PVS-QR | | CCTGCAGGGTCTAGGTAGACAG |  |
| PVX-QF | | GTACAGCTCGTGCCATAGTAG | primers for real time PCR |
| PVX-QR | | TGTGGGCACCTTCATGTCCTTC |  |
| PVM-QF | | CGAAGGGTGTGTAGGCTGTATG | primers for real time PCR |
| PVM-QR | | CAGCGAAGCGATCCTCATACT |  |

※Lowercase letters represent overlapping sequences

**Supplementary Table 2. Primers for constructing Y2H vectors.**

| **Primer name** | **Sequence (5'-3')** | **Note** |
| --- | --- | --- |
| StnCBP-BKF | ggccatggaggccgaattcATGGAAGTGACACCGGAGAAGA | to construct vector pGBKT7: StnCBP |
| StnCBP-BKR | ctgcaggtcgacggatccTTATGATCTTATCCAAGTATTGCGGTAAG |  |
| NbnCBP-BKF | ggccatggaggccgaattc ATGGAAGTGACAGCTGAGAAGAG | to construct vector pGBKT7: NbnCBP |
| NbnCBP-BKR | ctgcaggtcgacggatcc TTATCCTCTTATCCAAGTATTGCGGTATG |  |
| AtnCBP-BKF | ggccatggaggccgaattc ATGGAGGTTTTGGATAGGAGAGAC | to construct vector pGBKT7: AtnCBP |
| AtnCBP-BKR | ctgcaggtcgacggatcc CTATCCTCTCAGCCATGTGTTTC |  |
| SRaRp-ADF | atggaggccagtgaattc ATGGCACTTACTTACAGAAGTCCAA | to construct vector pGADT7: PVS RaRp |
| SRaRp-ADR | gctcgagctcgatggatcc TCAAACCTCCTCATACACACTGCG |  |
| STGB1-ADF | atggaggccagtgaattcATGAGGAGGTTTGACAGCTTAGGT | to construct vector pGADT7: PVS TGB1 |
| STGB1-ADR | gctcgagctcgatggatcc TTAGGCGGTGGTGTAAGTGG |  |
| STGB2-ADF | atggaggccagtgaattcATGCCACTTACACCACCGCC | to construct vector pGADT7: PVS TGB2 |
| STGB2-ADR | gctcgagctcgatggatcc TTAAGCACTGTGCACTCGATTGCA |  |
| STGB3-ADF | atggaggccagtgaattcATGTTGCCCAAGGTGCAATC | to construct vector pGADT7: PVS TGB3 |
| STGB3-ADR | gctcgagctcgatggatcc CTAAAGGTGTTTCAATGGCCTTAGTGTG |  |
| SCP-ADF | atggaggccagtgaattcATGCCGCCTAAACCAGATCCT | to construct vector pGADT7: PVS CP |
| SCP-ADR | gctcgagctcgatggatcc TCATTGGTTTGCTGCATTCCGAT |  |
| SNaBp-ADF | atggaggccagtgaattcATGAGAGCGGAACGTCTAAATATGTT | to construct vector pGADT7: PVS NaBp |
| SNaBp-ADR | gctcgagctcgatggatcc TCAGTTACTCCAACCTCGAATGAAAG |  |
| XRaRp-ADF | atggaggccagtgaattcATGGCCAAGGTGCGCGAG | to construct vector pGADT7: PVX RaRp |
| XRaRp-ADR | gctcgagctcgatggatcc TTAAAGAAAGTTTCTGAGGCGGGAA |  |
| XTGB1-ADF | atggaggccagtgaattcATGGATATTCTCATCAGTAGTTTGA | to construct vector pGADT7: PVX TGB1 |
| XTGB1-ADR | gctcgagctcgatggatcc CTATGGCCCTGCGCGGAC |  |
| XTGB2-ADF | atggaggccagtgaattcATGTCCGCGCAGGGCCAT | to construct vector pGADT7: PVX TGB2 |
| XTGB2-ADR | gctcgagctcgatggatcc CTAATGACTGCTATGATTGTTACCA |  |
| XTGB3-ADF | atggaggccagtgaattcATGGAAGTAAATACATATCTCAACG | to construct vector pGADT7: PVX TGB3 |
| XTGB3-ADR | gctcgagctcgatggatcc TCAATGGAAACTTAACCGTTCAACG |  |
| XCP-ADF | atggaggccagtgaattcATGTCAGCACCAGCTAGCAC | to construct vector pGADT7: PVX CP |
| XCP-ADR | gctcgagctcgatggatcc TTATGGTGGTGGTAGAGTGACAACA |  |
| MRaRp-ADF | atggaggccagtgaattcATGGCAGTCACATACAGAACGC | to construct vector pGADT7: PVM RaRp |
| MRaRp-ADR | gctcgagctcgatggatcc TTAAAGCACTTCAAATACTTGTTTCACA |  |
| MTGB1-ADF | atggaggccagtgaattcATGGATGTGATTGTAGATTTGTTGT | to construct vector pGADT7: PVM TGB1 |
| MTGB1-ADR | gctcgagctcgatggatcc TCAGGCGGCGGTGTAAGTGG |  |
| MTGB2-ADF | atggaggccagtgaattcATGCCACTTACACCGCCGCC | to construct vector pGADT7: PVM TGB2 |
| MTGB2-ADR | gctcgagctcgatggatcc TCATGTGTGTGAGCCCGCAC |  |
| MTGB3-ADF | atggaggccagtgaattcATGATAGTGTATGTACTTGTAGGACT | to construct vector pGADT7: PVM TGB3 |
| MTGB3-ADR | gctcgagctcgatggatcc TTATGACCTAAAGGAACCACACC |  |
| MCP-ADF | atggaggccagtgaattcATGGGTGATTCAACGAAGAAAGC | to construct vector pGADT7: PVM CP |
| MCP-ADR | gctcgagctcgatggatcc TCATTTTCTATTAGACTTTACATAATCTCT |  |
| MNaBp-ADF | atggaggccagtgaattcATGAAGGACGTAACCAAGGT | to construct vector pGADT7: PVM NaBp |
| MNaBp-ADR | gctcgagctcgatggatcc CTACTCTCGCTTGTTGATGACT |  |

**Supplementary Table 3. Primers for constructing subcellular localization and BiFC vectors.**

| **Primer name** | **Sequence (5'-3')** | **Note** |
| --- | --- | --- |
| StnCBP-GFPF | ctcggcatggacgagctgtacaag ATGGAAGTGACACCGGAGAAGA | to construct vector pK7WGF2: StnCBP |
| StnCBP-GFPR | cgggatatcaccactttgtacaTTATGATCTTATCCAAGTATTGCGGTAAG |  |
| SCP-GFPF | ctcggcatggacgagctgtacaag ATGCCGCCTAAACCAGATCCT | to construct vector pK7WGF2: NbnCBP |
| SCP-GFPR | cgggatatcaccactttgtaca TCATTGGTTTGCTGCATTCCGAT |  |
| StnCBP-RFPF | cgatatcacaagtttgtac ATGGAAGTGACACCGGAGAAGA | to construct vector pK7WGF2: AtnCBP |
| StnCBP-RFPR | ggatatcaccactttgtac TTATGATCTTATCCAAGTATTGCGGTAAG |  |
| StnCBP-Yn-F | actgttgatacatatgggatcc ATGGAAGTGACACCGGAGAAGA | to construct vector StnCBP-nYFP |
| StnCBP-Yn-R | taccgaattcactagtgtcgac TGATCTTATCCAAGTATTGCGGTAAG |  |
| NbnCBP-Yn-F | actgttgatacatatgggatcc ATGGAAGTGACAGCTGAGAAGAG | to construct vector NbnCBP-nYFP |
| NbnCBP-Yn-R | taccgaattcactagtgtcgac TCCTCTTATCCAAGTATTGCGGTATG |  |
| AtnCBP-Yn-F | actgttgatacatatgggatcc ATGGAGGTTTTGGATAGGAGAGAC | to construct vector AtnCBP-nYFP |
| AtnCBP-Yn-R | taccgaattcactagtgtcgac TCCTCTCAGCCATGTGTTTC |  |
| SCP-Yc-F | ctgttgatacatatgggatcc ATGCCGCCTAAACCAGATCCT | to construct vector PVS-CP-cYFP |
| SCP-Yc-R | accgaattcactagtgtcgac TTGGTTTGCTGCATTCCGAT |  |
| MCP-Yc-F | ctgttgatacatatgggatcc ATGGGTGATTCAACGAAGAAAGC | to construct vector PVM-CP-cYFP |
| MCP-Yc-R | accgaattcactagtgtcgac TCATTTTCTATTCGACTTTCCATAATCC |  |
| XCP-Yc-F | ctgttgatacatatgggatcc ATGTCAGCACCAGCTAGCAC | to construct vector PVX-CP-cYFP |
| XCP-Yc-R | accgaattcactagtgtcgac TTATGGTGGTGGTAGAGTGACAACA |  |

※Lowercase letters represent overlapping sequences

**Supplementary Table 4. Primers for constructing pCB301-2μ: PVS and pCB301-2μ: PVS^CP/M^, and pCB301-2μ: PVS^CP/X^**

| **Primer name** | **Sequence (5'-3')** | **Note** |
| --- | --- | --- |
| PVS-AJF | catttcatttggagaggCACTCCCGAAAATAATTTGACTTAAACAAC | to amplify fragment of PVS-A |
| PVS-AJR | GGCAAAGCTGCCTCAAAGTG |  |
| PVS-BJF | GTGCAGTCCATGGTTCTATACCAC | to amplify fragment of PVS-B |
| PVS-BJR | ACTACTAACACGCTCAAACTT |  |
| PVS-CJF | AGCTTAGGTAATCAGCTTAGTAGTAT | to amplify fragment of PVS-C |
| PVS-CJR | ggggaaattctttttttttttttttTTCTTTATAGTTGCACACTTATATATTATTTAAG |  |
| PVS-backboneF | ATAAGTGTGCAACTATAAAGAAaaaaaaaaaaaaaaagaatttccccgatcgttcaaac | to linearize plasmid pCB301-2μ-HDV |
| PVS-backboneR | GTTTAAGTCAAATTATTTTCGGGAGTGcctctccaaatgaaatgaacttcct |  |
| PVS/CP-backboneF | GAGCGGAACGTCTAAATATGTTAC | to linearize plasmid pCB301-2μ: PVS |
| PVS/CP-backboneR | TCTCTCTGTGAAACAGTTTATTCGA |  |
| M/CP-F | aactgtttcacagagaga ATGGGTGATTCAACGAAGAAAGC | to amplify fragment of PVM CP |
| M/CP-R | tatttagacgttccgctc TCATTTTCTATTCGACTTTCCATAATCC |  |
| X/CP-F | aactgtttcacagagaga ATGTCAGCACCAGCTAGCAC | to amplify fragment of PVM CP |
| X/CP-R | tatttagacgttccgctc TCATGGTGGTGGTAGAGTGACAACA |  |
| detection primer-F | AGCCGGAAGTCGGAGAGTTG | detection primers |
| detection primer-R | CAGATAGACAGATGAGCGCAACC |  |
| CP-flanking primer-F | CCACACTAAGGCCATTGAAACACCTTTA | CP-flanking primers |
| CP-flanking primer-R | CAGTCGGTAAACACACAGAAG |  |

※Lowercase letters represent overlapping sequences





**Supplementary Figure 1 Interference efficiency of RiStnCBP transgenic lines.** The gene expression was determined by qRT-PCR. Data are presented as means ± SD (n = 3) relative to WT (E3) plants, and *EF1α* was used as the normalizer. Asterisks indicate statistically significant differences according to Student’s t-test (p < 0.05 *, p < 0.01 **, p < 0.001 ***).


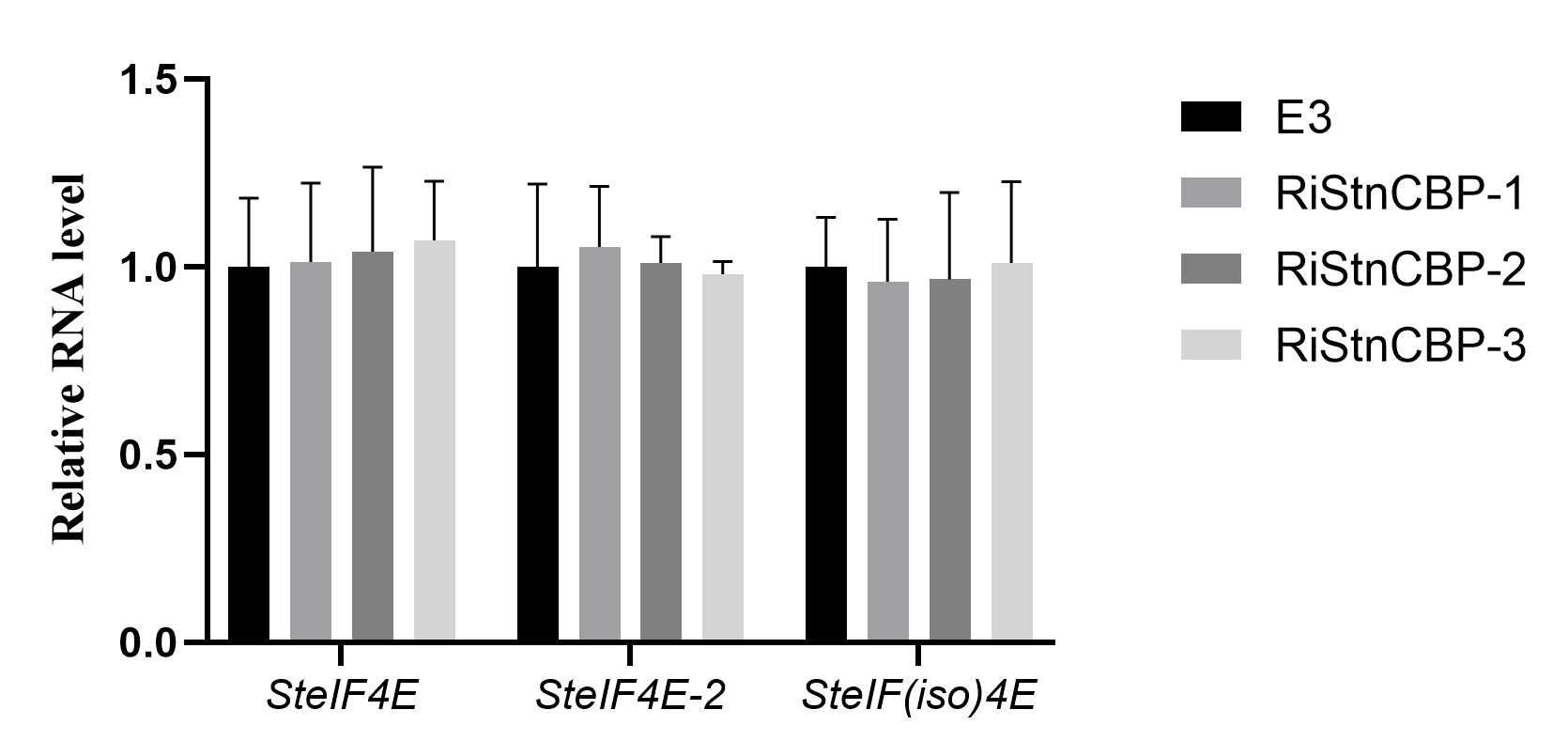


**Supplementary Figure 2 The expression of** ***SteIF4E*, *SteIF4E-2* and** ***SteIF(iso)4E* in the RiStnCBP lines and control plants (E3).** The gene expression was determined by qRT-PCR. Data are presented as means ± SD (n = 3) relative to WT (E3) plants, and *EF1α* was used as the normalizer.


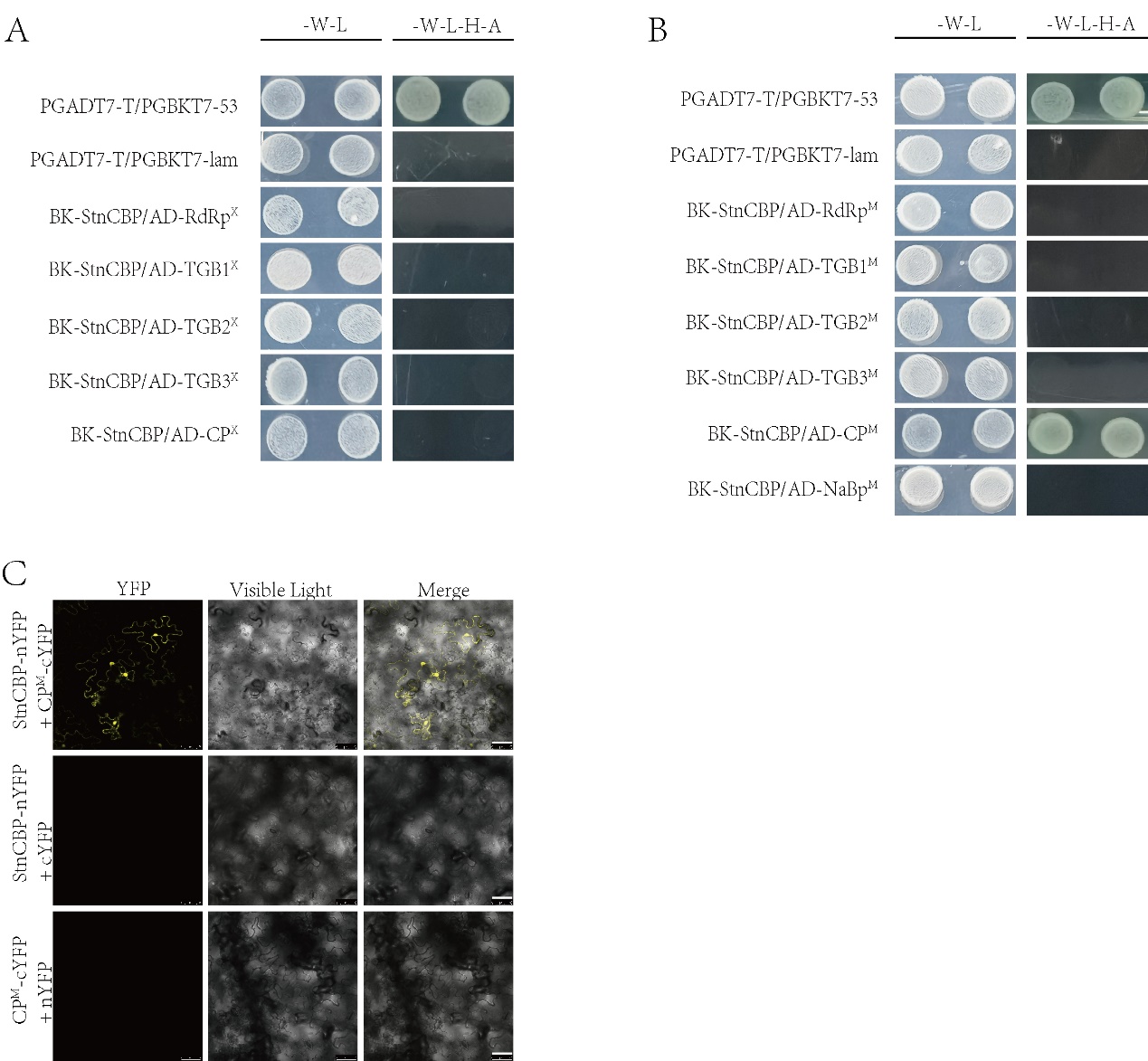


**Supplementary Figure 3 Interaction analysis of StnCBP with the proteins of PVX and PVM. (A)** Interaction between StnCBP and the proteins of PVX in Y2H assay. -W-L represents medium lacking tryptophan and leucine, -W-L-H-A represents medium lacking tryptophan, leucine, histidine, and adenine. Paired combinations PGADT7-T/PGBKT7-53 and PGADT7-T/PGBKT7-lam represent positive and negative controls, respectively. **(B)** Interaction between StnCBP and the proteins of PVM in Y2H assay. **(C)** Interaction of StnCBP with PVM CP in BiFC assay. Merge means the overlay of YFP and Visible Light on single confocal planes. Scale bar: 50 μm.


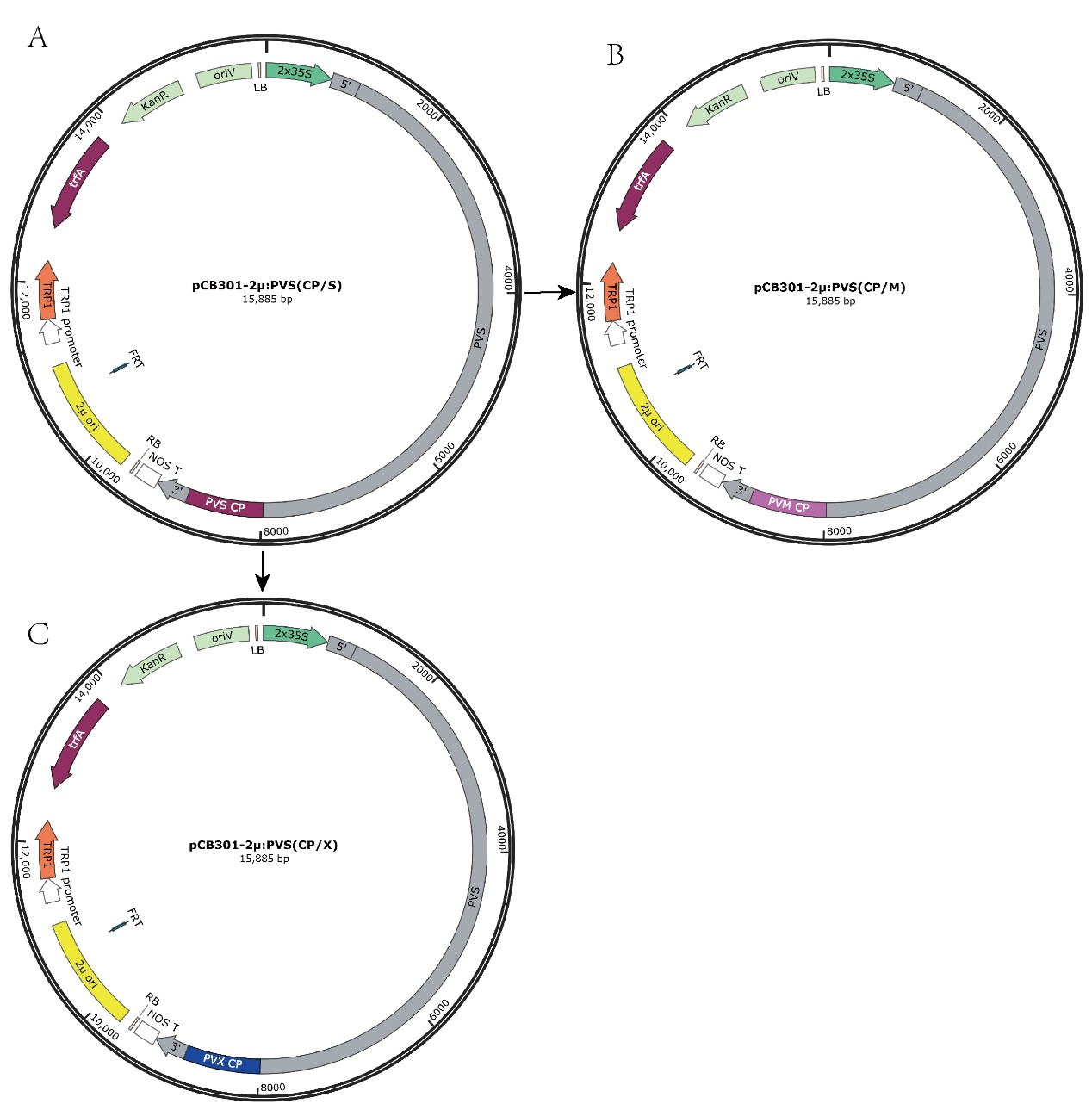


**Supplementary Figure 4 Schematic illustration shows the transformation of infectious clones PVS^CP/S^ (A) to PVS^CP/M^ (B) and** **PVS^CP/X^ (C).**

**
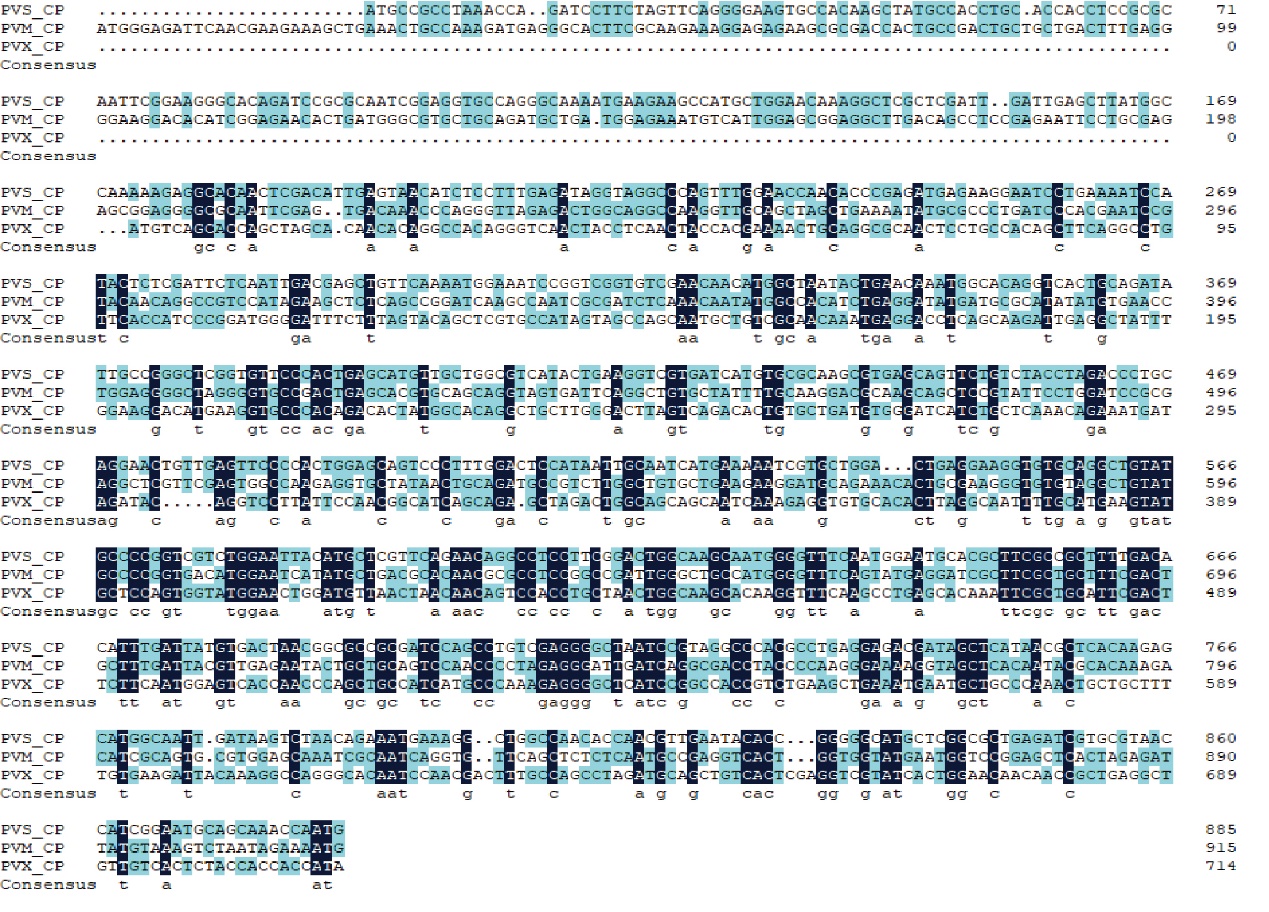
**

**Supplementary Figure 5 A nucleotide sequence alignment of the CPs from PVS, PVM and PVX.** The nucleotide lengths of CP from PVS, PVM, and PVX are 885bp, 915bp, and 714bp, respectively.


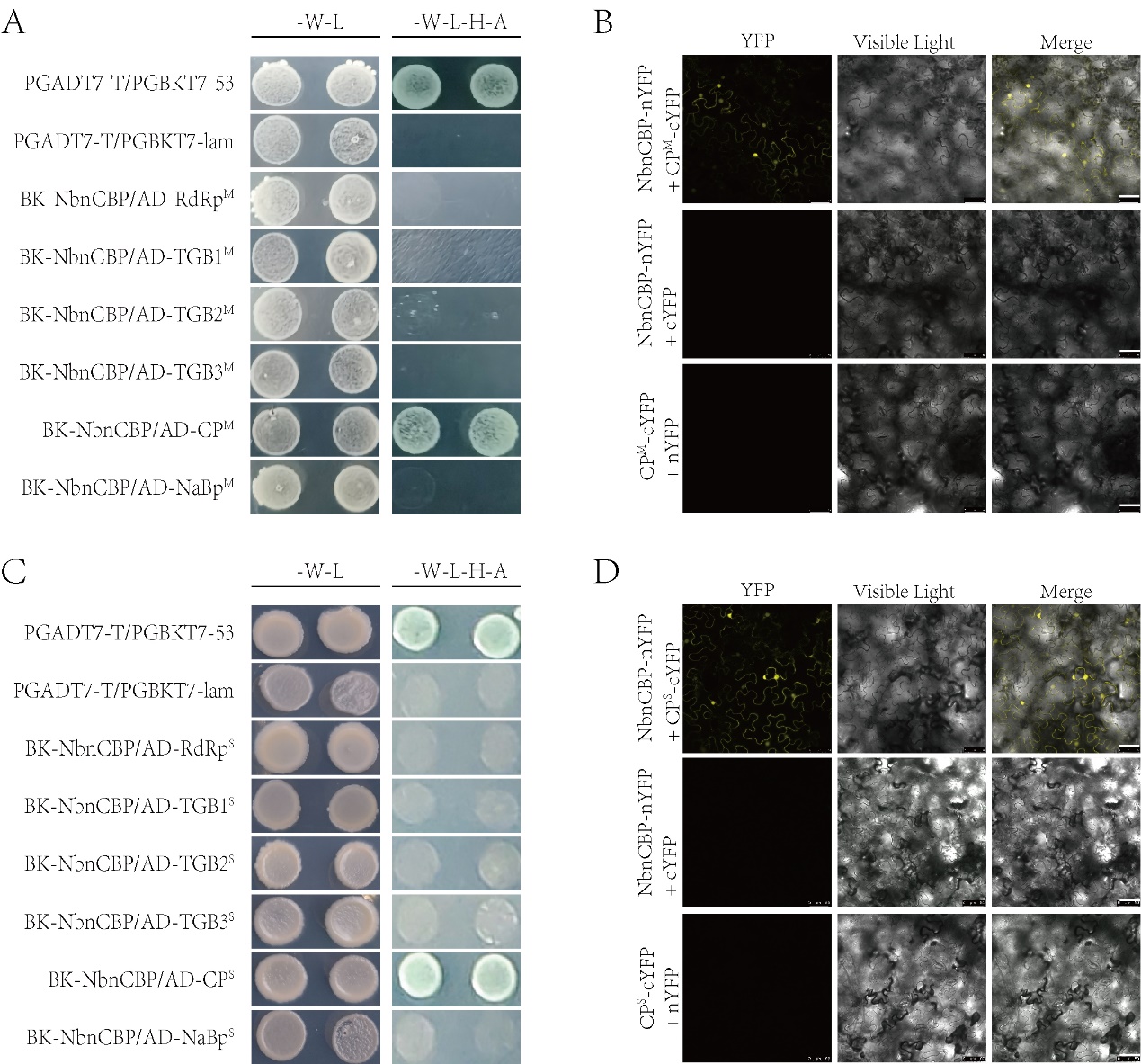


**Supplementary Figure 6 Interaction analysis between NbnCBP and the proteins of PVM and PVS. (A)** Interaction between NbnCBP and proteins of PVM in Y2H assays. **(B)** Interaction between NbnCBP and PVM CP in BiFC assays. **(C)** Interaction between NbnCBP and proteins of PVS in Y2H assays. **(D)** Interaction between NbnCBP and PVS CP in BiFC assays. In Y2H assays, -W-L represents medium lacking tryptophan and leucine, -W-L-H-A represents medium lacking tryptophan, leucine, histidine, and adenine. Paired combinations PGADT7-T/PGBKT7-53 and PGADT7-T/PGBKT7-lam represent positive and negative controls, respectively. In BiFC assays, Merge means the overlay of YFP and Visible Light on single confocal planes. Scale bar: 50 μm.


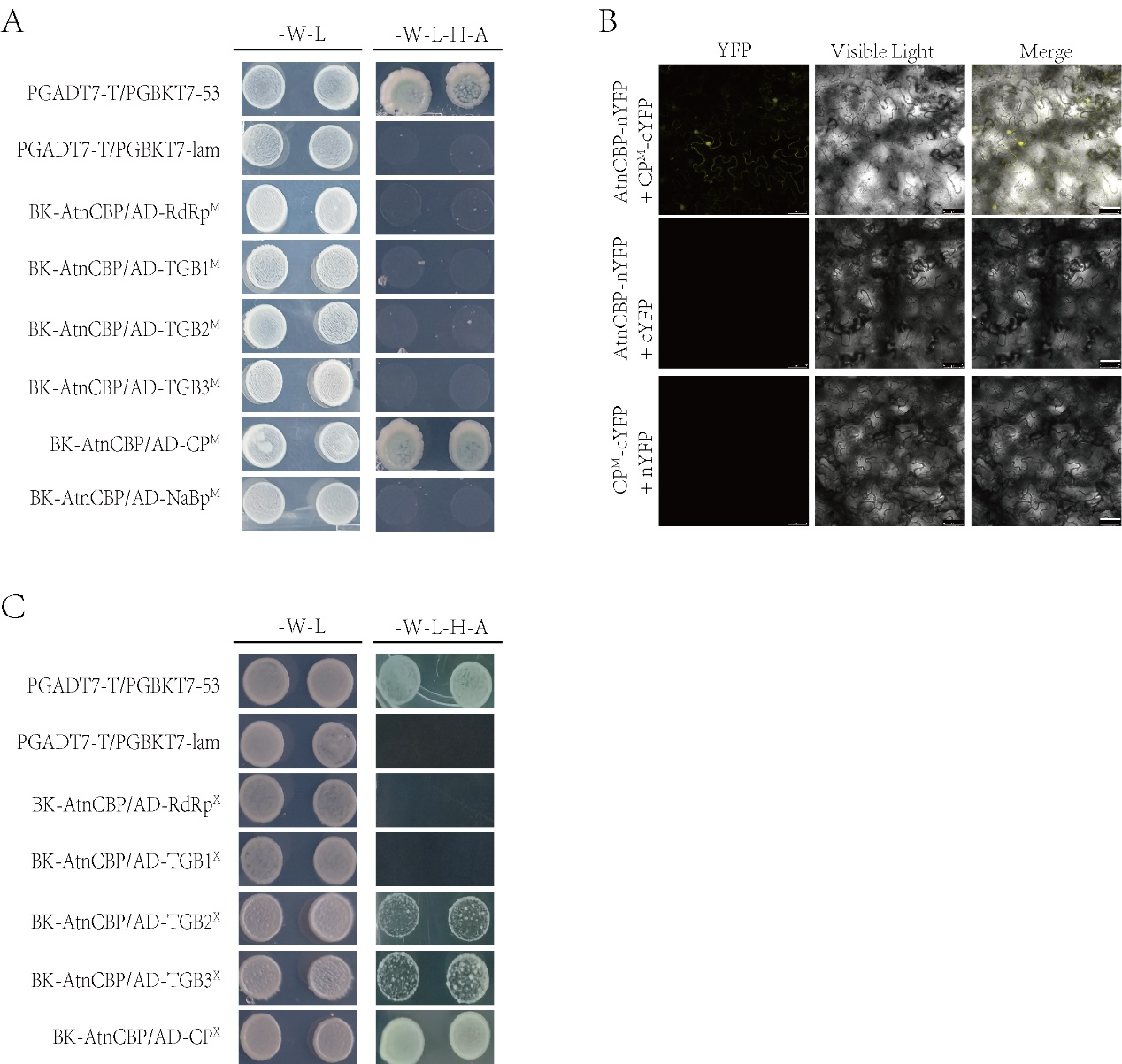


**Supplementary Figure 7 Interaction analysis of AtnCBP with the proteins of PVM and PVX**

**(A)** Interaction between AtnCBP and proteins of PVM in Y2H assays. **(B)** Interaction between NbnCBP and PVM CP in BiFC assays. Merge means the overlay of YFP and Visible Light on single confocal planes. Scale bar: 50 μm. **(C)** Interaction between AtnCBP and proteins of PVX in Y2H assays. -W-L represents medium lacking tryptophan and leucine, -W-L-H-A represents medium lacking tryptophan, leucine, histidine, and adenine. Paired combinations PGADT7-T/PGBKT7-53 and PGADT7-T/PGBKT7-lam represent positive and negative controls, respectively.
